# Intracellular lipid droplets positively regulate the dormancy of colorectal cancer cells depending on ELF1-ACSL5 signaling

**DOI:** 10.1186/s13046-026-03754-y

**Published:** 2026-06-11

**Authors:** Wancheng Liao, Rui Yin, Fan Zuo, Yangkun Li, Jiao Deng, Yanqi Li, Xiaolan Li, Deding Tao, Xuelai Luo, Weike Ji, Jichao Qin

**Affiliations:** 1 Molecular Medicine Center, Tongji Hospital, Tongji Medical College, Huazhong University of Science and Technology, Wuhan, Hubei 430030 China; 2 Department of Surgery, Tongji Hospital, Tongji Medical College, Huazhong University of Science and Technology, Wuhan, Hubei 430030 China; 3 Department of Gastrointestinal Surgery, the First Affiliated Hospital, Zhejiang University School of Medicine, Hangzhou, Zhejiang 310003 China; 4 Department of Biochemistry and Molecular Biology, School of Basic Medicine, Tongji Medical College and State Key Laboratory for Diagnosis and Treatment of Severe Zoonotic infectious Diseases, Huazhong University of Science and Technology, Wuhan, Hubei 430030 China; 5 Cell Architecture Research Center, Huazhong University of Science and Technology, Wuhan, Hubei 430030 China

**Keywords:** Lipid droplets, Dormant cancer cells, Tumor recurrence

## Abstract

**Background:**

Colorectal cancer recurrence is largely attributed to dormant tumor cells that evade therapy. The role of organelle metabolism, particularly lipid droplets (LDs) accumulation, in maintaining tumor dormancy remains poorly understood. This study aimed to investigate how LDs contribute to dormancy and to identify key regulatory mechanisms.

**Methods:**

Using patient-derived xenograft models and colorectal cancer cell lines, we established chemotherapy-induced dormant, proliferative, and recurrent cells. Transcriptomic profiling identified key regulatory genes and lipidomics of LDs was performed by Liquid Chromatography‑Tandem Mass Spectrometry. Functional validation was performed via genetic knockdown/overexpression, pharmacological inhibition, and mutational analysis. Lipid droplet content, reactive oxygen species levels, and dormancy markers were assessed using staining, flow cytometry, and immunoblotting. Clinical correlation was evaluated using patient datasets and survival analysis.

**Results:**

Dormant cells exhibited elevated LD levels compared to proliferative or recurrent cells. Transcriptomic analysis identified ACSL5 as a key upregulated gene in dormant cells. ACSL5 promoted LD accumulation, which in turn reduced intracellular reactive oxygen species and stabilized dormancy by sequestering oxidized lipids. The transcription factor ELF1 directly bound the ACSL5 promoter, establishing the ELF1-ACSL5 regulatory axis. Disruption of this axis depleted LDs, increased ROS, and forced dormant cells to exit dormancy. Clinically, high ACSL5 expression correlated with poor prognosis in colorectal cancer patients.

**Conclusion:**

This study defines a novel ELF1-ACSL5-lipid droplet axis that maintains colorectal cancer dormancy by promoting lipid sequestration and redox homeostasis. These findings reveal organelle-centric metabolic reprogramming as a fundamental dormancy mechanism and nominate this axis as a promising therapeutic target to prevent cancer recurrence.

**Supplementary Information:**

The online version contains supplementary material available at 10.1186/s13046-026-03754-y.

## Background

Colorectal cancer (CRC) is a leading malignancy globally [1, 2], yet recurrence remains a major clinical challenge [3, 4]. Tumor relapse is mechanistically linked to tumor cell dormancy [5], a clinically silent state where quiescent cancer cells evade therapy and immune surveillance [6]. In CRC, dormant cells can persist after initial treatments like surgery and chemotherapy [5], retaining the potential to reactivate and cause disease recurrence. This transition into dormancy involves complex signaling pathways. Understanding the link between dormancy and recurrence is therefore crucial for developing strategies to prevent relapse and improve patient outcomes.

Under microenvironmental stressors such as hypoxia, nutrient deprivation, or oxidative stress, dormant tumor cells exhibit substantial LD accumulation, with the precise mechanism remaining elusive [7]. Once viewed as simple energy stores, LDs are now recognized as dynamic organelles composed of neutral lipids within a phospholipid monolayer [8]. Metabolic reprogramming in dormant cells—characterized by suppressed glycolysis and enhanced fatty acid oxidation (FAO)—paradoxically increases reactive oxygen species (ROS) generation, thereby inducing oxidative stress [9, 10]. The oxidative burden is further exacerbated by pro-oxidant metabolites (e.g., peroxides, hydroperoxides) produced during fatty acid metabolism [11, 12]. However, dormant tumor cells require low levels of ROS to maintain their quiescent state [13]. LDs counteract this by sequestering oxidized lipids, such as peroxidized PUFAs, thereby reducing ROS and cellular damage—a protective mechanism observed in astrocytes [14]. Furthermore, LDs interact directly with the endoplasmic reticulum (ER) via lipid transport proteins, helping to regulate ER lipid metabolism and mitigate ER stress [15]. The role and mechanistic basis of accumulated lipid droplets (LDs) in regulating tumor cell dormancy remain unknown.

This study explores both the regulation of LD accumulation in dormant CRC cells and its functional impact on maintaining dormancy. Using cell lines and patient-derived xenograft models, we demonstrate that the ELF1-ACSL5 axis directly regulates LD content in dormant CRC cells. This LD accumulation reduces intracellular ROS levels, thereby reinforcing dormancy maintenance.

## Methods

### Cell culture

LoVo colorectal carcinoma cells and HEK293T embryonic kidney cells (ATCC, USA) were maintained in DMEM (Gibco #12800, USA) supplemented with 10% fetal bovine serum (F101, Vazyme, China) and 1% penicillin-streptomycin (Thermo Fisher #15140-122, USA) at 37 °C under 5% CO₂.

Generated from patient-derived xenografts via enzymatic dissociation [16, 17]. Tumor fragments were digested at 37 °C for 30 min in DMEM/F12 (Thermo Fisher, USA) containing collagenase IV (1.5 mg/mL), hyaluronidase (20 µg/mL; Sigma-Aldrich, USA), and penicillin-streptomycin (100 U/mL).

Single-cell suspensions were enriched via EpCAM-BUV737 labeling (BD Biosciences, USA) and sorted using a FACS Aria II (BD Biosciences). Clinical and cell line details are provided in Supplementary Table S1. Drugs, antibodies, and kits used in this study see Table S2.

### EdU assays

PDX1 and LoVo cells were cultured in DMEM (Gibco) supplemented with 10% fetal bovine serum (FBS) and 1% penicillin-streptomycin at 37 °C in a humidified 5% CO₂ atmosphere. Cells (1 × 10⁴ per well) were seeded onto coverslips in 96-well plates and incubated until reaching 60–70% confluency. EdU (10 µM; Abbkine, KTA2031) was added to the culture medium for 2 h. Cells were fixed with 4% paraformaldehyde (25 min, room temperature), washed with PBS, and permeabilized with 0.5% Triton X-100 (20 min, room temperature). For Click-iT detection, fixed cells were incubated with the Click-iT reaction cocktail (30 min, room temperature, protected from light). Nuclei were counterstained with DAPI (0.1 µg/mL, 5 min).

### Dual luciferase assay

The ACSL5 promoter region was amplified using primers (Table S3) and genomic DNA extracted with the TaKaRa MiniBEST Universal Genomic DNA Extraction Kit (Ver. 5.0; Takara Bio Inc., #9765). Fragment size was confirmed by 1% agarose gel electrophoresis. The pGL3-Basic vector (Addgene, #48743) was linearized by PCR and ligated to the ACSL5 promoter fragment using the ClonExpress Ultra One Step Cloning Kit (Vazyme, C116) to generate the pGL3-ACSL5 promoter construct. The pRL-TK plasmid (Beyotime, D2760), encoding Renilla luciferase, served as an internal control. HEK293T cells were co-transfected with the pGL3-ACSL5 promoter construct, pRL-TK, and an ELF1 overexpression plasmid using HighGene Plus Transfection Reagent (ABclonal, RM09014P). After 48 h, cells were lysed, and lysates were mixed with luciferase substrate (Beyotime, RG027). Reactions were transferred to solid white 96-well plates (Beyotime, FCP968) to minimize light scattering. Luminescence was quantified using a Varioskan LUX microplate reader (Thermo Scientific). Firefly luciferase activity was normalized to Renilla luciferase. Statistical analysis was performed using GraphPad Prism 9.0. Primers for the dual luciferase assay are listed in Table S4.

### Plasmids and shRNAs

shRNAs targeting ACSL5 (P53216, P53220, P53240), ELF1 (P45344, P45342, P45341), FIP200 (P85385, P85475, P85470), DGAT1 (P85508, P85390, P85382), DGAT2 (P85496, P85378, P85375) were obtained from MiaoLingPlasmid, China. Overexpression plasmids for ACSL5 (P59918, P59843) and ELF1 (P55614) were obtained from MiaoLingPlasmid, China. The construct was utilized following excision of the corresponding GFP sequence. pRL-TK (D2760) Plasmids was purchased from Beyotime, China. pGL3-Basic (#48743) Plasmids was purchased from Addgene.

### Lentivirus construction and transduction

400 µL of Opti-MEM (Gibco), 7.5 µg of psPAX2 plasmid (#12260, Addgene), 2.5 µg of pMD2.G plasmid (Addgene), 10 µg of shRNA plasmids (MiaoLingBio), and 20 µL of HighGene plus Transfection reagent (RM09014P, ABclonal) were combined and added to HEK293T cells. Lentiviral particles were harvested from HEK293T cell supernatants 48–72 h post-transfection, clarified by centrifugation (2,000 × g), and sterilized via 0.22-µm filtration. Viral supernatants were then applied to target cells for transduction. Post-infection, cells underwent 16-hour viral exposure followed by puromycin selection (2 µg/mL; Thermo Fisher Scientific, USA) initiated 48 h post-transduction, with sustained antibiotic pressure maintained for 5 days. Successful transduction was verified through immunoblot analysis of target protein expression.

### Sphere-forming assay

1 × 10⁵ tumor cells were cultured in ultra-low-attachment 6-well plates (Corning, #3471, USA) with 2 mL of sphere medium: DMEM/F12 (Gibco, USA) supplemented with B27 (1:50 dilution; Invitrogen, #17504044, USA), recombinant human FGF (20 ng/mL; Invitrogen, #PHG0263), recombinant human EGF (20 ng/mL; Invitrogen, #PHG0311), heparin (4 mg/mL; Sigma-Aldrich, #H3149, USA), and 1% penicillin-streptomycin (Thermo Fisher, #15140-122, USA). Spheres were harvested every 5 days by centrifugation (300 × g, 5 min), dissociated into single-cell suspensions using 0.25% trypsin-EDTA (Thermo Fisher, #25200056, 10 min at 37 °C), counted with a hemocytometer, and re-plated at the original density.

### Immunofluorescence

For in vitro imaging, cells were cultured on 24-well glass coverslips (1 × 10⁵ cells/well) for 24 h. After PBS rinsing, samples were fixed with 4% paraformaldehyde (RT, 30 min), permeabilized with 0.4% Triton X-100/5% BSA in PBS (15 min), and subjected to sequential antibody incubation: primary antibodies (4 °C, 12 h) followed by fluorophore-conjugated secondary antibodies (RT, 2 h). Nuclei were counterstained with DAPI (Sigma-Aldrich, USA) for 5 min, and slides were mounted with antifade reagent (Thermo Fisher Scientific, USA) for imaging on Olympus BX53/CKX41 systems. Clinical immunofluorescence analyses were performed in partnership with Fiber Biont (Wuhan, China), utilizing matched colorectal carcinoma (CRC) tissues, hepatic metastatic lesions, and adjacent normal specimens from CRC patients. The clinical history of the human subject was listed in Supplementary Table S5. For subcellular localization of total lipids and peroxidized lipids in tumor cells. Live cells were stained with BODIPY C11 (red) for 30 min at 37 °C, fixed, and then counterstained with DAPI for 5 min prior to fluorescence microscopy imaging. Upon oxidation, this probe exhibits a spectral shift in fluorescence emission from 590 nm (red) to 510 nm (green).

### Western blot

Cells were washed three times with cold PBS. Cellular proteins were lysed in NP-40 buffer containing protease inhibitor cocktail (HY-K0010, MedChemExpress, USA) for 30 min at 4 °C. Protein concentrations were quantified using a BCA assay kit (#23225, Thermo Fisher Scientific, USA). Subsequently, lysates underwent electrophoretic separation on SDS-PAGE gels and electrotransfer to PVDF membranes. Membranes were blocked with 5% skim milk, then probed with primary antibodies at 4 °C for 12 h. Following incubation with HRP-conjugated secondary antibodies (1:5000 dilution, room temperature, 2 h), immunoreactive bands were detected via chemiluminescence substrate (#34096, Thermo Fisher Scientific).

### Flow cytometry

Flow cytometry data were acquired by using a FACS Aria II Cell Sorter (BD Biosciences, San Jose, CA, USA), followed by flow cytometric analysis using Diva software (BD Biosciences), Flowjo or Novo Express.

The lipid droplets amounts in the cell lines were measured using flow cytometry. The indicated cell lines were seeded in six-well plates at a density of 5 × 10^5^ cells per well before the experiment. Following experimental interventions, cells were collected and stained with 5 µM BODIPY 493/503 (HY-W090090, MedChemExpress, USA) in PBS at 37 °C for 30 min. After washing and resuspension in 500 µL PBS, fluorescence signals were analyzed on a BD FACSuite flow cytometer with 488 nm excitation.

For ROS quantification, pre-seeded cells (3 × 10⁵ cells/well in 6-well plates) were treated as described. Post-treatment samples were incubated with 25 × 10⁶ M DCFH-DA (S0033, Beyotime, China) in PBS under identical staining conditions (37 °C, 30 min), followed by flow cytometric analysis using the same instrument parameters.

### Cell viability assay

Cell proliferation was evaluated with the Cell Counting Kit-8 (CCK-8; HY-K0301, MedChem Express, USA). LoVo and PDX1 cell lines were cultured in 96-well plates (5 × 10³ cells/well) and exposed to experimental compounds. At specified intervals, 10 µL of CCK-8 reagent was introduced into each well. Absorbance values were recorded at 450 nm after 2.5 h of incubation.

### Cell cycle assay

For cell cycle profiling, DNA content distribution was analyzed via flow cytometry using propidium iodide staining. After trypsinizing and collecting the cells, wash the cells in PBS to remove debris. Fixed in precooled 75% ethanol. Ethanol was added dropwise to the cell pellet during vortexing. This ensures that all cells are fixed, minimizing cell aggregation. Fix at 4 °C for 30 min. Wash 2 times with PBS. Centrifuge the cells at 850 g, care should be taken to avoid loss of cells when discarding the supernatant, especially when centrifuging to remove ethanol. Cells are treated with ribonucleases. Add 50 µL of RNase stock solution (100 µg/mL). This ensures that only DNA is stained, not RNA. Add 200 µL of PI (50 µg/mL of storage solution). Detect the cell cycle using a flow cytometer.

### Reverse transcription quantitative (RT-q) PCR analysis

Total RNA was isolated with TRIzol reagent (Takara Bio Inc., Japan) following the manufacturer’s protocol, followed by cDNA synthesis using the HiScript III 1st Strand cDNA Synthesis Kit (R312-02, Vazyme, China). Quantitative PCR amplifications were conducted with ChamQ Universal SYBR qPCR Master Mix (Q711-02, Vazyme, China) on an ABI 7300 QuantStudio 3 system. Primer pairs specific to target genes are documented in Supplementary Table S6.

### Bulk RNA sequencing (RNA-seq)

Following cell seeding into 6-well plates and pharmacological treatment, the supernatant was aspirated and cells were washed three times with pre-cooled PBS buffer. Cellular lysis was subsequently performed using Trizol reagent (Takara Bio Inc., Japan). Subsequent sequencing procedures were conducted by Major Biotechnology Corporation (Shanghai, China) following standard protocols. The raw sequencing data underwent comprehensive bioinformatics analysis including quality control, read alignment, and differential gene expression profiling using established pipelines.

### Site-directed mutagenesis

Based on established reports of ACSL5 functional inactivation, we introduced the clinically relevant missense mutation NM_016234.4:c.1526 C > A (p.Thr509Lys) into the coding sequence. Mutation-specific primers were designed using the Vazyme Single-Point Mutagenesis Primer Design Platform (https://tool.vazyme.com:18002/cetool/singlepoint.html). Site-directed mutagenesis was performed according to the manufacturer’s protocol for the Mut Express™ Universal Fast Mutagenesis Kit (Vazyme, C216-01). The resulting plasmid constructs were transformed into DH5α competent *E. coli* cells, followed by Sanger sequencing verification of the targeted mutation. The primers for site-directed mutagenesis are provided in Supplementary Table S7.

### Acyl-CoA synthetase activity assay

Cellular ACS activity was quantified using the Amplex™ Red Acetyl-CoA Synthetase Assay Kit (Beyotime, S0391S). Adherent cells were washed with PBS and lysed with 100–200 µl of ice-cold BeyoLysis™ Buffer A for Metabolic Assay (Beyotime) per 1 × 10^6^ cells. After gentle pipetting and 10-min incubation on ice, lysates were centrifuged at 12,000 × g for 5 min at 4 °C. Supernatants were collected for immediate analysis. The reaction working solution was prepared according to the kit protocol, containing 100 µM Amplex Red reagent, 2 U/mL horseradish peroxidase, 0.2 U/mL acetate kinase, 1 mM acetyl-CoA, and 1 mM oxaloacetate in assay buffer. A standard curve was generated using 0–10 µM CoA standards. Samples (50 µl) were mixed with 50 µl working solution and incubated at 37 °C for 30 min in the dark. Fluorescence intensity was quantified using a Varioskan LUX multimode microplate reader (Thermo Scientific) with excitation at 560 nm and emission at 590 nm.

### Endoplasmic reticulum isolation

Endoplasmic reticulum (ER) fractions were isolated using the Endoplasmic Reticulum Extraction Kit (Solarbio, EX1370). Cells were pelleted by centrifugation (500 × g, 5 min, 4 °C), resuspended in Reagent A, and incubated on ice for 10 min. Homogenization was performed with a Dounce homogenizer (30–40 strokes). The lysate was centrifuged at 1,000 × g for 5 min (4 °C), and the supernatant was collected. Subsequent centrifugation at 11,000 × g for 10 min (4 °C) yielded a supernatant that was ultracentrifuged at 50,000 × g for 45 min (4 °C). The resulting pellet was resuspended in 400 µL ice-cold Reagent B, mixed thoroughly, and recentrifuged (50,000 × g, 45 min, 4 °C). The final ER-enriched pellet was resuspended in ER storage buffer and stored at -80 °C for downstream applications.

### Mitochondrial isolation

Mitochondria were isolated using the Mitochondria Isolation Kit (Beyotime, C3601). Cells were detached by trypsinization, washed with PBS, and gently resuspended in mitochondrial isolation reagent. After 10-min incubation on ice, cells were homogenized with 20 strokes in a Dounce homogenizer. The homogenate was centrifuged at 600 × g for 10 min (4 °C). The supernatant was transferred to a new tube and centrifuged at 11,000 × g for 10 min (4 °C). The resulting pellet containing enriched mitochondria was resuspended in storage buffer for downstream applications.

### Lipid droplet isolation and lipidomic analysis

#### Lipid droplet isolation

Lipid droplets (LDs) were isolated using the Lipid Droplet Isolation Kit (Cell Biolabs, MET-5011). Cells were washed twice with PBS, detached by trypsinization, and pelleted by centrifugation (500 × g, 5 min). The pellet was resuspended in 200 µL ice-cold Reagent A and incubated on ice for 10 min. After adding 800 µL of 1× Reagent B, the suspension was mixed thoroughly and incubated on ice for 10 min. Homogenization was performed by passing the lysate five times through a 27-gauge needle attached to a 3-mL syringe. The homogenate was centrifuged at 100 × g for 5 s to remove debris. A discontinuous gradient was formed by carefully layering 600 µL of 1× Reagent B atop the supernatant. Samples were ultracentrifuged at 20,000 rpm for 3 h at 4 °C. The top LD-containing fraction (270 µL) was collected and transferred to RNase-free microcentrifuge tubes.

#### Lipid extraction and LC‑MS/MS analysis

For lipid droplet (LD) isolation, frozen samples were thawed at 4 °C. An aliquot of 100 µL was freeze-dried, then resuspended in 190 µL of 10 mM ammonium formate in butanol‑methanol (1:1, v/v). After adding 10 µL of internal standard, the mixture was vortexed for 30 s, sonicated at room temperature for 1 h, and centrifuged at 13,000 × g for 10 min. The supernatant was collected for analysis. For whole‑cell lysates, cells were thawed at 4 °C and 400 µL of the same ammonium formate‑butanol‑methanol solution were added. The mixture was vortexed, sonicated, and centrifuged as above. Then, 40 µL of the supernatant were mixed with 150 µL of the ammonium formate solution and 10 µL of internal standard, and the sample was ready for injection. Chromatographic separation was performed on a Nexera X2 LC‑30AD ultra‑high‑pressure liquid chromatography system (Shimadzu). Mobile phase A consisted of 50% acetonitrile containing 10 mM ammonium acetate (pH 8.0), and mobile phase B was 100% acetonitrile. The gradient elution program was: 0–0.1 min, 90% B; 0.1–5 min, linear decrease from 90% to 65% B; 5–5.3 min, linear decrease from 65% to 0% B; 5.3–7.2 min, hold at 0% B; 7.2–7.4 min, linear increase from 0% to 90% B; 7.4–10 min, hold at 90% B. The column temperature was maintained at 35 °C, the flow rate was 300 µL/min, and the injection volume was 1 µL. Three injections were performed per sample using different mass spectrometry methods. Mass spectrometry was carried out on a Triple Quad 7500 mass spectrometer (AB SCIEX) operating in both positive and negative ion modes. The source parameters were: ionspray voltage, + 5500/‑4500 V; temperature, 400 °C; ion source gas 1, 55 psi; ion source gas 2, 60 psi; curtain gas, 35 psi; collision gas, 8 (arbitrary units). Declustering potential (DP) and collision energy (CE) were optimized for each lipid class individually. Peak areas were extracted and integrated using Multiquant software. Lipids were identified and quantified by comparing retention times with authentic lipid standards, using an internal standard method. The concentration (X) was calculated as X = (R × MIS)/V, where R is the peak area ratio of the sample to the internal standard, MIS is the amount of internal standard added, and V is the sample volume used. All statistical and bioinformatic analyses were performed with R software. Lipid nomenclature followed the guidelines of the LIPID MAPS consortium [Liebisch et al., J. Lipid Res. 2020, doi:10.1194/jlr.S120001025]. In multiple reaction monitoring (MRM), the exact positions of acyl chains cannot always be determined; underscores (e.g., PC(16:0_18:1)) indicate uncertain positional assignment. For triacylglycerols, “TG 54:3‑FA18:1” denotes a total chain length of 54 carbons with three double bonds, where the fatty acid at the sn‑3 position is likely oleic acid (18:1); the other two positions may contain various fatty acid combinations. Lipidomic profiling of purified LDs and whole cell lysis (Medical high-throughput targeted lipid panel (absolute quantification)) was conducted with the help of Bioprofile Biological Technology Co., Ltd. (Shanghai).

### Animal experiments

All experimental procedures involving animals were reviewed and approved by the Animal Welfare and Ethics Committee of Tongji Hospital, Tongji Medical College, Huazhong University of Science and Technology (Approval No. TJH-202408072). All mice were purchased from Beijing Vital River Laboratory Animal Technology Co., Ltd. All mice were housed in the Animal Experiment Center of the Scientific Research Building at Tongji Hospital, Tongji Medical College, Huazhong University of Science and Technology. Upon arrival, mice underwent a one-week quarantine period in the facility to confirm good health status before being transferred to the designated housing rooms for subsequent experiments. Mice were maintained under specific pathogen-free (SPF) barrier conditions, with ad libitum access to standard laboratory rodent chow and autoclaved water. All personnel involved in animal experiments had completed the Hubei Province Laboratory Animal Operation Training and passed the assessment to obtain professional qualification certificates. Operations were conducted in strict accordance with the requirements of the Animal Welfare and Ethics Committee. For surgical procedures, including tumor cell implantation, mice were anesthetized with 2% isoflurane delivered in oxygen via a precision vaporizer. At the experimental endpoint, mice were euthanized by cervical dislocation. All efforts were made to minimize animal suffering.

Female BALB/c nude mice (4 weeks old) were used to generate the subcutaneous tumor model. CRC cells were collected and washed twice with PBS. Cells were resuspended in a 1:1 (v/v) PBS/Matrigel mixture, followed by injection into the subcutaneous tissue using a 27-gauge needle. After 2 months, Tumors were monitored, and tumor volumes were examined every three days. After sacrifice, tumors were removed from mice and weighed to evaluate the tumor development. For limiting dilution assays, tumor-initiating frequency and statistical significance were evaluated with the Extreme Limiting Dilution Analysis (ELDA) ‘limdil’ function (http://bioinf.wehi.edu.au/software/elda/index.html).

### Patients and subject details

The Ethics Committee of Tongji Hospital granted ethical approval for this study (TJ-IRB20230934). Clinical samples were sourced from the Department of Gastrointestinal Surgery at Tongji Hospital. Demographic characteristics (age and sex distribution) are summarized in Supplementary Table S1 and S5.

### Statistical analysis

Heatmap generation. Lipidomics data were log₂‑transformed and row‑scaled (Z‑score). For each lipid species (row), the Z‑score was calculated as Z = (x – µ)/σ, where x represents the log₂‑transformed abundance, µ is the mean across all samples, and σ is the standard deviation. This scaling emphasizes relative changes across groups rather than absolute abundance differences. Heatmaps were generated using the pheatmap package (version 1.0.12) in R (version 4.2.1). The color palette ranged from blue (low abundance) to orange (high abundance), with white representing the mean (Z‑score = 0). Analytical procedures were executed using GraphPad Prism 9.0 (GraphPad Software, CA, USA). Continuous variables are expressed as mean ± standard deviation. Experimental replicates (n-values) for each analysis are detailed in corresponding figure captions. Survival probabilities were evaluated through Kaplan-Meier methodology with log-rank comparative testing. Correlation assessments employed Pearson’s linear coefficient and Spearman’s rank-based analysis. Intergroup comparisons utilized Student’s two-sample t-test (two-sided) and one-way ANOVA with Tukey’s post-hoc adjustment. Statistical thresholds were defined as follows: *p < 0.05, **p < 0.01, ***p < 0.001, ****p < 0.0001.

## Results

### Establishing the dormant and recurrent colorectal cancer cells in vitro

To model tumor heterogeneity, we established a patient-derived xenograft (PDX1) from a surgical CRC specimen. Our prior work demonstrated that PDX models faithfully recapitulate parental tumor heterogeneity [18], making PDX1 our primary model.

**Fig. 1:**
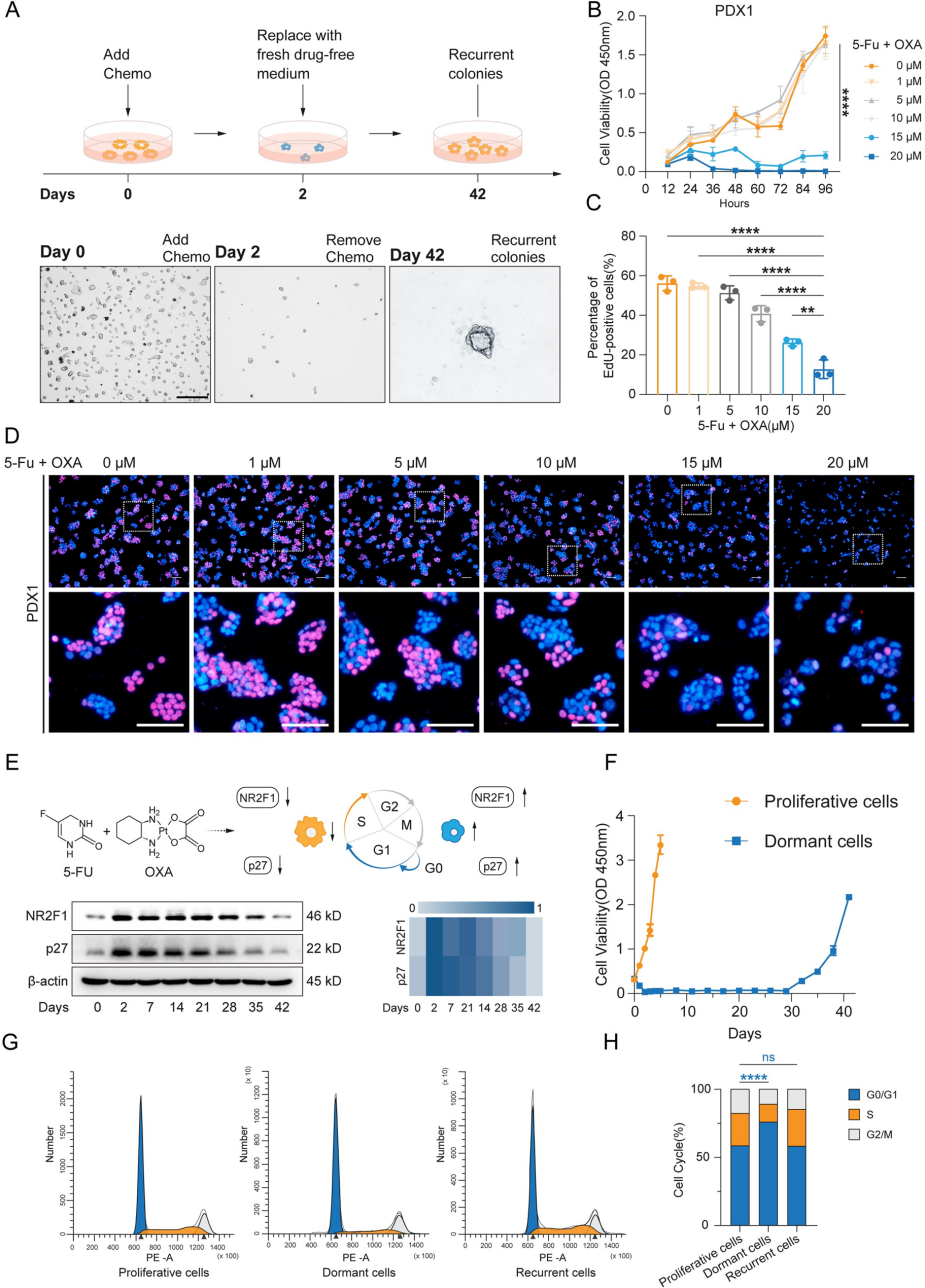
Establishing the dormant and recurrent colorectal cancer cells in vitro **A** Schematic workflow for generating dormant and recurrent CRC cells: CRC cells were cultured in the medium containing chemotherapeutic agents (5-Fu + OXA) for 2 days, followed by replacement with regular medium and continued to be cultured until recurrent colonies emerged at day 42. Brightfield pictures of dormant and recurrent PDX1 cells were shown below. Scale bar: 100 μm. **B** Cell viability (OD 450 nm) of the PDX1 cells treated with the indicated doses of 5-Fu and OXA for 96 h was detected by CCK-8 assays (n = 3). **C**-**D** The PDX1 cells treated with the indicated doses of 5-Fu and OXA for 48 h was detected by EdU assay (n = 3). D Representative microphotographs. Scale bar: 20 μm. **E** Western blot analysis of NR2F1 and p27 expression in the PDX1 cells that originated from (**C**) at the indicated time. **F** Cell viability of the PDX1 cells that originated from (**C**) at the indicated time by CCK-8 assay (n = 3). **G**-**H** Cell cycle analysis for the proliferative, dormant and recurrent PDX1 cells by flow cytometry. **G** Representative image of the cell cycle flow cytometry. **H** The statistical analysis of the cell-cycle analysis. Shown are the mean percentages of cells in different phases of the cell cycle (n = 3). Data are presented as mean ± SD. All error bars in all figures represent the mean ± SD unless otherwise stated. Statistical analysis was performed using one-way ANOVA in (**C** and **H**), and two-way ANOVA in (**B**). ns, no significance. **p < 0.01, ****p < 0.0001

Residual dormant cancer cells are implicated in tumor recurrence among CRC patients undergoing radical resection [19, 20]. Therapy-induced dormancy, driven by chemoresistance, is a key barrier to preventing metastatic relapse [21, 22]. Preclinical models employing therapeutic perturbations (e.g., in vitro and in vivo systems) are widely used to mimic this dormant phenotype, reflecting quiescent subpopulations observed in post-treatment patients [22]. To model dormancy and recurrence in vitro, CRC cells were treated for 48 h with 5-fluorouracil (5-Fu) and oxaliplatin (OXA)—standard chemotherapeutics for advanced CRC—at varying concentrations [23]. After drug washout, cells were maintained in drug-free medium (Fig. 1A). Combination treatment with 15 µM or 20 µM 5-Fu and OXA halted cellular proliferation, whereas lower concentrations (0–10 µM) did not, establishing 15–20 µM as the dormancy induction threshold (Fig. 1B and Supplementary Fig. S1A). EdU staining confirmed a marked reduction in proliferating cells at these concentrations (Fig. 1C-D). Following 48-hour treatment with 15 µM 5-Fu/OXA, the majority of PDX1 cells underwent apoptosis, while a residual subpopulation survived. This residual population exhibited no detectable proliferation over subsequent days (data not shown). After 40 days of drug withdrawal, residual cells resumed proliferation, forming clonal colonies (Fig. 1A), indicative of dormancy exit and transition to recurrence.

To assess dormancy exit, we performed western blotting and CCK-8 assays following chemotherapeutic withdrawal. Dormancy markers, NR2F1 and p27 [24], were highly expressed immediately post-treatment but declined progressively over 42 days in drug-free medium (Fig. 1E). CCK-8 assays confirmed proliferative arrest during treatment, with resumption of growth 28 days after drug-free medium replacement (Fig. 1F). Cell cycle analysis via flow cytometry further corroborated dormancy induction. Treatment with 5-Fu/OXA increased G0/G1-phase arrest, while pharmacological withdrawal shifted cells to S/G2-M phases (Fig. 1G-H). Collectively, our model demonstrates that CRC cells enter therapy-induced dormancy under 5-Fu/OXA exposure and re-enter the cell cycle upon drug withdrawal.

### Dormant CRC cells are endowed with more lipid droplets than proliferative and recurrent cells

**Fig. 2:**
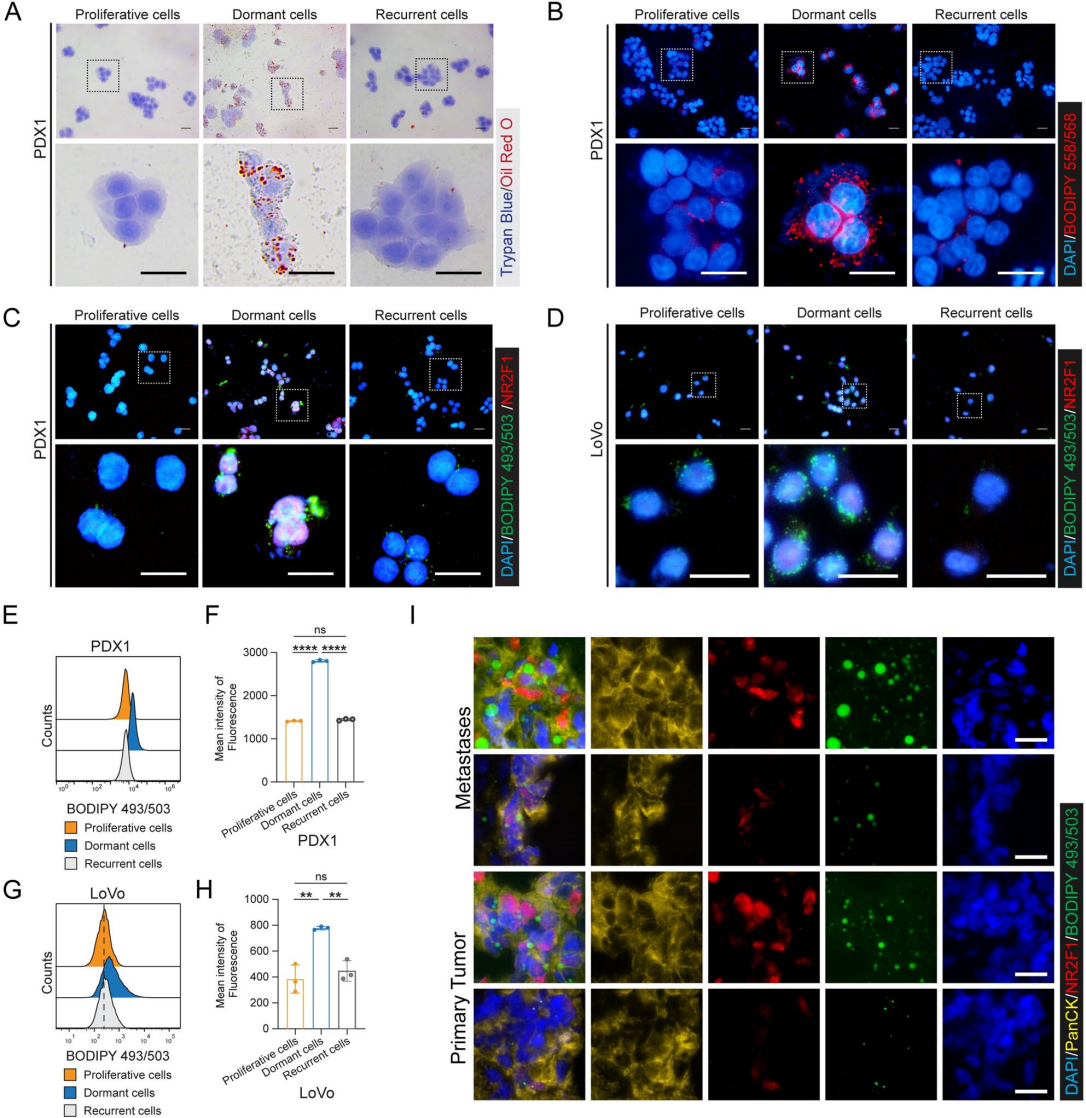
Dormant CRC cells are endowed with more lipid droplets than proliferative and recurrent cells. **A**-**B** Representative microphotographs of the lipid droplets in proliferative, dormant and recurrent PDX1 cells stained by Oil red O or BODIPY 558/568. Lipid droplets (Oil red O/BODIPY 558/568, red) and nucleus (Trypan Blue/DAPI, blue). Scale bar: 20 μm. **C**-**D** Representative microphotographs of the immunofluorescence staining for the lipid droplets in proliferative, dormant and recurrent PDX1 and LoVo cells. NR2F1 (red), lipid droplets (BODIPY 493/503, green) and nucleus (DAPI, blue). Scale bar: 20 μm. **E**-**H** The lipid droplets contents in proliferative, dormant and recurrent PDX1 and LoVo cells were measured by Flow cytometry. (F and H) The statistical analysis of the flow cytometry (n = 3). **I** Representative multiplex immunofluorescence images of CRC tissues stained for PanCK (yellow; cancer cells), NR2F1 (red; dormancy marker), BODIPY 493/503 (green; lipid droplets), and DAPI (blue; nuclei). Scale bar: 20 μm. Data are presented as mean ± SD. Statistical analysis was performed using one-way ANOVA in (**F** and **H**). ns, no significance. **p < 0.01, ****p < 0.0001

In pancreatic ductal adenocarcinoma (PDAC), dormant cells exhibit greater LDs abundance compared to proliferating counterparts, as LDs serve as energy reservoirs to sustain survival during dormancy [7]. To assess LD accumulation across cell states, we first employed Oil Red O (ORO) staining in dormant, proliferative, and recurrent CRC cells. ORO staining is a well-established method for visualizing neutral lipid content in cellular systems [25]. Dormant cells displayed significantly higher LD levels than proliferative or recurrent cells (Fig. 2A). Consistent results were obtained using BODIPY 558/568, a fluorescent dye widely utilized for LD visualization (Fig. 2B) [26]. NR2F1 (nuclear receptor subfamily 2, group F, member 1), a transcriptional regulator of tumor cell dormancy [24, 27, 28], was co-stained with LDs to interrogate their relationship. Co-staining for NR2F1 and LDs demonstrated that dormant CRC cells, which exhibited elevated NR2F1 expression, accumulated substantially more LDs than proliferative or recurrent cells (Fig. 2C-D). Flow cytometry quantification further confirmed elevated LD content in dormant PDX1 (Fig. 2E-F) and LoVo (Fig. 2G-H) cells compared to proliferative or recurrent populations. We performed analyses on a cohort of four post-chemotherapy and three chemotherapy-naïve CRC patient samples (see Supplementary Material Table 5 for details) (Fig. 2I and Supplementary Fig. S1B). Anti-PanCK (a cancer cell-specific epithelial marker) was used to distinguish tumor cells from stromal cells. Histopathological analysis of patient-derived CRC specimens (primary tumors, hepatic metastases, and adjacent non-tumoral tissues) indicated that dormant tumor cells in both primary and metastatic lesions contained more lipid droplets (LDs) than proliferating cells (Fig. 2I). Quantification across all four post-chemotherapy samples revealed that, on average, 82% of PanCK⁺ cancer cells were double-positive for NR2F1 and LDs (Supplementary Fig. S1B). Collectively, these findings establish that dormant CRC cells consistently accumulate higher LD levels than proliferative or recurrent populations across experimental and clinical models.

### The expression of ACSL5 is dynamically related to the dormant state of colorectal cancer cells

**Fig. 3:**
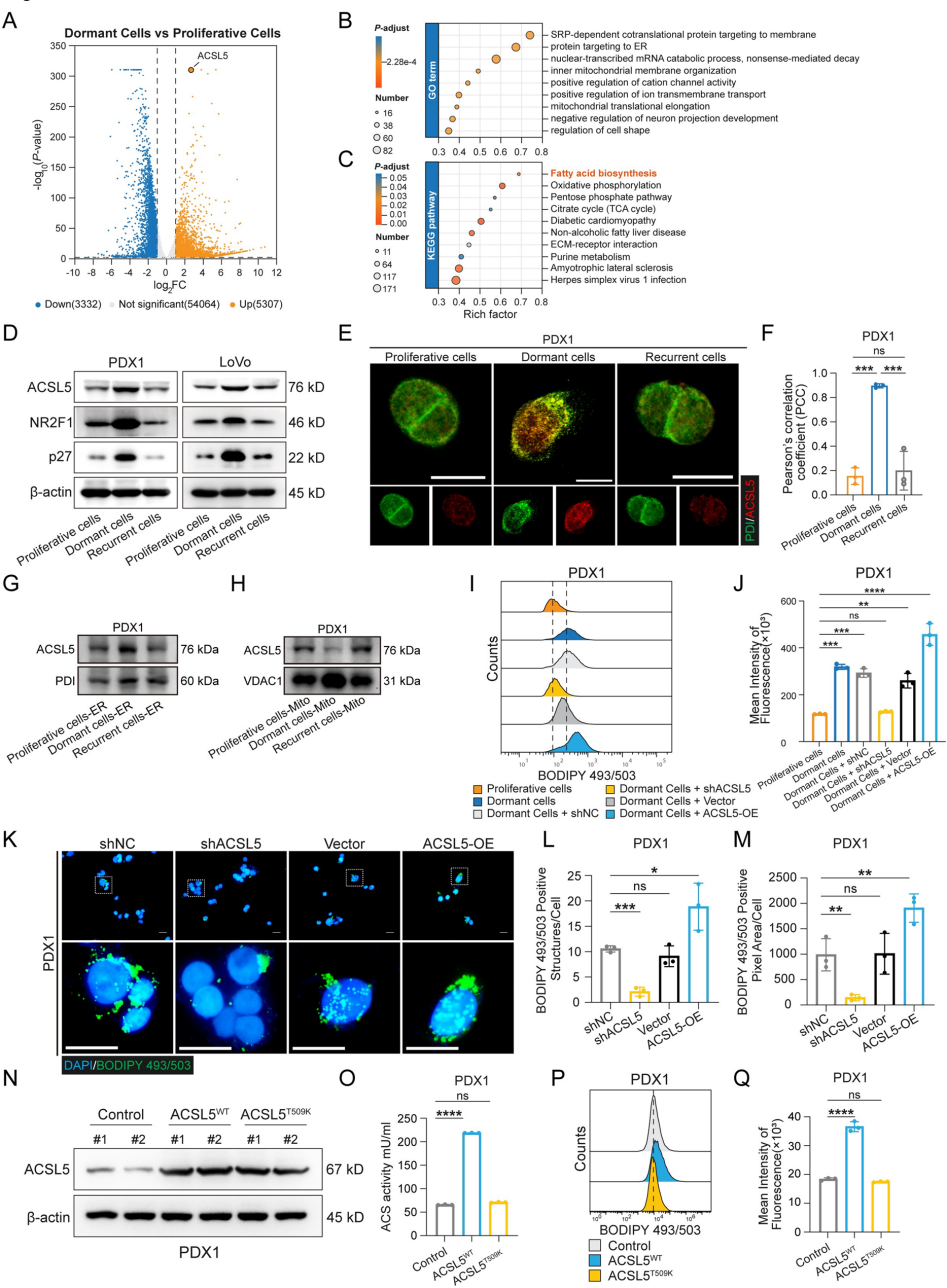
ACSL5 positively regulates lipid droplet abundance in dormant CRC cells. **A** Volcano plot displaying differentially expressed genes (DEGs) in dormant vs proliferative PDX1 cells. **B**-**C** GO and KEGG enrichment analysis of DEGs in dormant vs proliferative PDX1 cells. **D** Western blot analysis of ACSL5 in proliferative, dormant and recurrent PDX1 and LoVo cells. **E** Immunofluorescence staining of ACSL5 (red) and ER (PDI, green). Scale bar: 20 μm. **F** Pearson's correlation coefficient (PCC) was used to assess the degree of co-localization of ACSL5 and endoplasmic reticulum in proliferative, dormant, and recurrent PDX1 cells (n = 3). **G**-**H** Western blot analysis of ACSL5 levels in endoplasmic reticulum or mitochondrial fractions isolated from proliferative, dormant, and recurrent PDX1 cells. Raw blots are shown in the Supplementary Materials. **I**-**J** The lipid droplets were measured in dormant PDX1 cells upon knockdown or overexpression of ACSL5 by flow cytometry (n = 3). **K** Representative microphotographs of immunofluorescence staining to observe the lipid droplets in dormant PDX1 cells upon knockdown or overexpression of ACSL5. Scale bar: 20 μm. **L**-**M** The statistical analysis of abundance or area of lipid droplets in Figure 3K (n = 3). **N** Western blot analysis confirming expression levels of wild-type ACSL5 (ACSL5^WT^) and the catalytic dead point mutant (ACSL5^T509K^) in PDX1 cells. **O** ACS enzymatic activity assay measuring acyl-CoA synthetase (ACS) activity in cells expressing vector control, ACSL5^WT^, and ACSL5^T509K^ in PDX1 cells (n = 3). Activity is shown in mU/ml. **P**-**Q** Flow cytometric analysis of lipid droplet content in tumor cells expressing vector control, ACSL5^WT^, and ACSL5^T509K^ in PDX1 cells. **Q** The statistical analysis of the flow cytometry (n = 3). Data are presented as mean ± SD. Statistical analysis was performed using one-way ANOVA in (F, J, L-M, O and Q). ns, no significance. *p < 0.05, **p < 0.01, ***p < 0.001, ****p < 0.0001

To interrogate the regulation of LDs content, we performed bulk RNA sequencing (RNA-seq) on dormant, recurrent, and proliferative CRC cells. Dormant tumor cells exhibit a distinct transcriptional profile compared to their proliferative and recurrent counterparts (Fig. 3A and Supplementary Fig. S2A). LDs originate from the endoplasmic reticulum (ER), as established in prior studies [11]. Gene ontology (GO) analysis revealed that upregulated genes in dormant cells were enriched in pathways such as translational initiation and endoplasmic reticulum protein localization (Fig. 3B). KEGG pathway analysis and literature mining highlighted upregulated fatty acid biosynthesis genes in dormant cells, among which *ACSL5*, *ACSL6*, *ACSBG1*, and *ACSBG2* emerged as the top candidate genes mechanistically linked to LD biogenesis and lipid metabolism [11, 29, 30] (Fig. 3C). *ACSL5* and *ACSL6* belong to the acyl-CoA synthetase long-chain (ACSL) family, which catalyzes long-chain fatty acid activation to acyl-CoA for lipid synthesis or β-oxidation [29]. Similarly, *ACSBG1* and *ACSBG2*, members of the bubblegum acyl-CoA synthetase family, regulate lipid metabolism [30]. Other genes within this pathway, including FASN, were downregulated at the mRNA level in dormant cells, suggesting that they may not play a critical role in our experimental system. To validate transcriptional and translational expression of these candidates, we performed qRT-PCR and western blotting. At the mRNA level, all four genes were significantly upregulated in dormant cells compared to proliferative and recurrent populations (Supplementary Fig. S2C-F). At the protein level, however, only ACSL5 was upregulated, while ACSL6, ACSBG1, and ACSBG2 were reduced in dormant cells (Fig. 3D and Supplementary Fig. S2G), nominating ACSL5 as a key regulator of LD abundance in dormant CRC cells.

ACSL5 is a recognized prognostic marker in CRC, with established roles in tumorigenesis and progression [31–33]. Compartment-specific studies have shown that ER-localized ACSL5 promotes triglyceride synthesis and LD formation, while mitochondrial ACSL5 facilitates β-oxidation for energy production [29, 34, 35]. The ER serves as the primary site for LD biogenesis [11, 36]. We therefore investigated the subcellular localization of ACSL5 across different cell states. Immunofluorescence revealed predominant ER localization of ACSL5 in dormant cells (Fig. 3E-F), whereas recurrent cells exhibited increased mitochondrial ACSL5 (Supplementary Fig. S2H-I), suggesting compartment-specific roles in LD regulation. Subcellular fractionation confirmed ACSL5 enrichment in the ER and lower levels in mitochondria of dormant cells (Fig. 3G-H and Supplementary Fig. S2J-K). Collectively, these findings demonstrate that ACSL5 is highly expressed and predominantly ER-localized in dormant CRC cells, where it drives intracellular LD accumulation during tumor dormancy.

### ACSL5 positively regulates the abundance of LDs in dormant CRC cells

To determine ACSL5’s role in LDs regulation, we performed ACSL5 knockdown and overexpression in PDX1 and LoVo cells (Supplementary Fig. S3A-B). ACSL5 knockdown significantly reduced LD content, while its overexpression increased LD levels in dormant cells, as quantified by flow cytometry (Fig. 3I-J and Supplementary Fig. S3C-D) and immunofluorescence (Fig. 3K-M and Supplementary Fig. S3E-G). Collectively, these data establish ACSL5 as a critical positive regulator of LD accumulation in dormant CRC cells.

ACSL5 catalyzes free fatty acids into acyl-CoA esters, a critical step for triacylglycerol synthesis [37]. To investigate how ACSL5 mediates elevated LD content in dormant tumor cells, we first inhibited its enzymatic activity using the ACSL5-specific inhibitor Triacsin C [38], which significantly suppressed LD accumulation in dormant cells (Supplementary Fig. S3H-K). Subsequently, we generated an ACSL5 loss-of-function point mutant (T509K) [39], which ablates ATP binding and eliminates enzymatic activity without affecting protein expression, as confirmed by immunoblotting (Fig. 3N and Supplementary Fig. S3L). ACS activity assays verified near-complete loss of enzymatic function in the mutant (Fig. 3O and Supplementary Fig. S3M). Critically, this catalytic inactivation of ACSL5 abrogated fatty acid activation and markedly inhibited LD accumulation in tumor cells (Fig. 3P-Q and Supplementary Fig. S3N-O). Collectively, these data demonstrate that ACSL5 drives intracellular LD accumulation in dormant tumor cells by facilitating fatty acid activation and subsequent triacylglycerol (TAG) synthesis.

### ELF1 transcriptionally regulates ACSL5 expression in dormant CRC cells

**Fig. 4:**
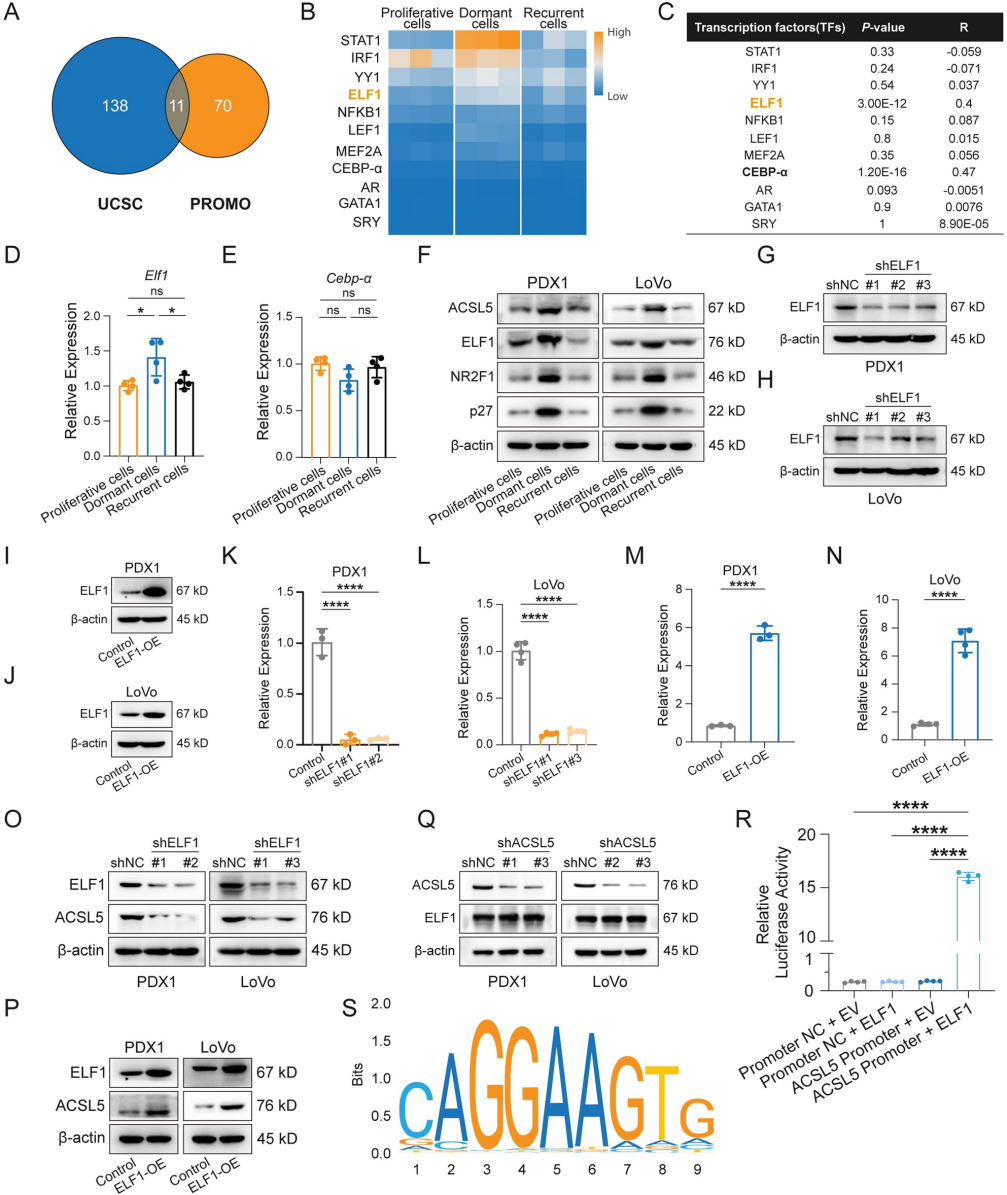
ELF1 transcriptionally regulates ACSL5 expression in dormant CRC cells.**A** The transcriptional factors (TFs) analysis of ACSL5 using UCSC and PROMO databases. **B** Heat map presentation of candidate TFs of ACSL5 in the aforementioned transcriptome sequencing dataset. **C** Quantitative assessment of Pearson correlation coefficients between these TFs and ACSL5 expression. Values of *R* < 0 denote inverse (negative) correlations, whereas *R* > 0 reflect direct (positive) correlations. Data derived from the GEPIA2 database, with statistical significance indicated. **D-E** qPCR analysis of *ELF1* and *C/EBP-α* mRNA levels in proliferative, dormant and recurrent PDX1 cells (*n* = 4). **F** Western blot analysis of ELF1 expression in proliferative, dormant and recurrent PDX1 and LoVo cells. **G-J** Validation of the knockdown and overexpression of ELF1 in PDX1 and LoVo cells. **K-N** qPCR analysis of ACSL5 mRNA levels in PDX1 and LoVo cells following ELF1 knockdown or overexpression (*n* = 3). **O-P** Western blot analysis of ACSL5 expression upon knockdown or overexpression of ELF1 in PDX1 and LoVo cells. **Q** Western blot analysis of ELF1 expression upon knockdown of ACSL5 in PDX1 cells. **R** Luciferase assay validating ELF1 transcriptionally regulates ACSL5 expression in dormant CRC cells (*n* = 4). **S** The predicted binding motif of ELF1 on the promoter sequence of ACSL5 by JASPAR database. Data are presented as mean ± SD. Statistical analysis was performed using one-way ANOVA in (D-E, K-N and R). ns, no significance. *p < 0.05, **p < 0.01, ***p < 0.001, ****p < 0.0001

To identify transcriptional regulators of ACSL5 in dormant CRC cells, we analyzed its promoter region (–1950 to + 50 bp) using UCSC and PROMO databases [35], predicting 11 candidate binding transcription factors (TFs) (Fig. 4A) with expression shown in a heatmap (Fig. 4B). Correlation analysis via GEPIA2 indicated ELF1 and C/EBP-α as top candidates associated with ACSL5 expression (Fig. 4C). qRT-PCR confirmed that ELF1 mRNA levels, but not C/EBP-α, aligned with RNA-seq data (Fig. 4D and E). Western blot analysis revealed elevated ELF1 protein expression in dormant versus proliferative or recurrent CRC cells (Fig. 4F).

ELF1, an ETS-family transcription factor implicated in cell cycle regulation, DNA repair, and epithelial-mesenchymal transition (EMT) [40], was functionally interrogated. ELF1 knockdown reduced, while its overexpression increased, both ACSL5 mRNA and protein levels (Fig. 4G-P). ACSL5 depletion did not alter ELF1 expression (Fig. 4Q), confirming ELF1’s upstream position in this regulatory axis. Luciferase reporter assays confirmed enhanced ACSL5 promoter activity under ELF1 overexpression (Fig. 4R-S). Collectively, these data establish ELF1 as a direct transcriptional activator of ACSL5 in dormant CRC cells.

### The ELF1-ACSL5 axis positively regulates lipid droplets content of the dormant CRC cells

**Fig. 5:**
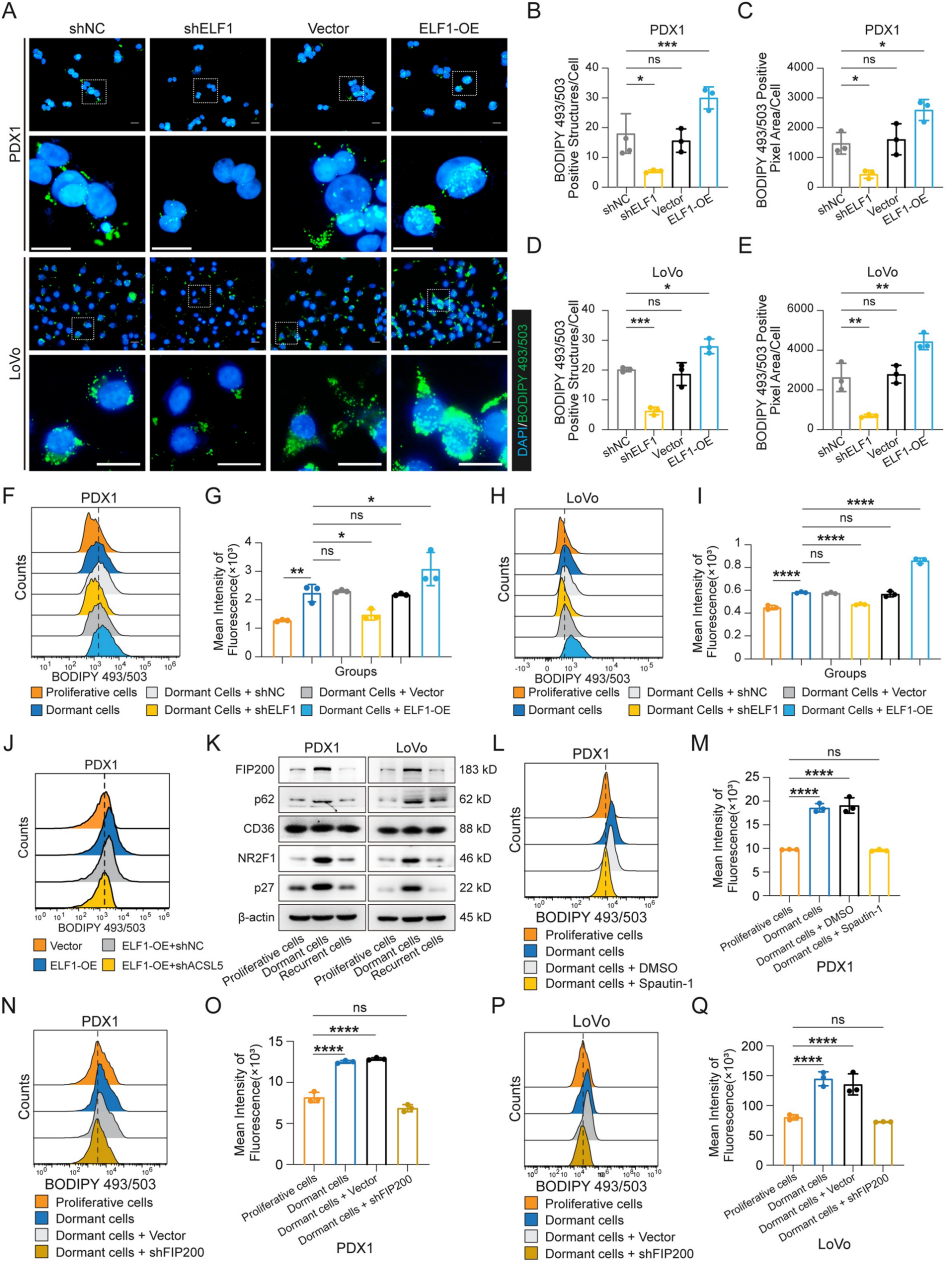
The ELF1-ACSL5 axis positively regulates lipid droplets content of the dormant CRC cells.** A-E **Fluorescence staining to detect the lipid droplets in dormant PDX1 and LoVo cells upon knockdown or overexpression of ELF1.** A **Representative microphotographs. Scale bar: 20 μm. **B**-**E**. The statistical analysis of the abundance or area of lipid droplets (n = 3). **F**-**I** The lipid droplets were measured in dormant PDX1 and LoVo cells upon knockdown or overexpression of ELF1 by flow cytometry. Shown in (**G** and **I**) are the results of the statistical analysis (n = 3). **J** The lipid droplets were examined by flow cytometry in PDX1 cells overexpressed ELF1 and/or knocked down ACSL5. **K** Western blot analysis of key autophagy pathway components and lipid transporters in proliferative, dormant, and recurrent PDX1 and LoVo cells. **L**-**M** Flow cytometric analysis of lipid droplet content in dormant PDX1 cells following autophagy inhibition with Spautin-1. **M** The statistical analysis of the flow cytometry (n = 3). **N**-**Q** Flow cytometric analysis of lipid droplet content in chemotherapy-induced dormant tumor cells following FIP200 knockdown in PDX1 and LoVo cells. **O** and **Q** The statistical analysis of the flow cytometry (n = 3). Data are presented as mean ± SD, and statistical analysis was performed using one-way ANOVA. ns, no significance. *p < 0.05, **p < 0.01, ***p < 0.001, ****p < 0.0001

To determine whether the ELF1-ACSL5 axis regulates LDs content in dormant CRC cells, we performed BODIPY 493/503 staining after modulating ELF1 expression. ELF1 knockdown reduced LD abundance, whereas ELF1 overexpression increased it (Fig. 5A-I), demonstrating that ELF1 positively regulates LD accumulation in dormant CRC cells. To dissect this axis, we overexpressed ELF1 with or without ACSL5 knockdown in dormant CRC cells. ELF1 overexpression enhanced LD levels, an effect reversed by ACSL5 knockdown (Fig. 5J and Supplementary Fig. S4A-C), confirming that ELF1 regulates LD accumulation through ACSL5 in dormant CRC cells.

We next investigated the lipid source supporting LD biogenesis. Although exogenous lipid assimilation may contribute, enhanced autophagy in dormant cells emerged as the primary candidate [10]. Immunoblot analysis showed elevated autophagic flux (FIP200, p62) but unchanged CD36 expression in dormant cells (Fig. 5K). We re‑analyzed our RNA‑seq data to examine other lipid transporters and found that FABP1 and FABP6 were downregulated in dormant cells, whereas FABP3 and FABP5P9 were upregulated (Supplementary Fig. S4D). Furthermore, a functional lipid uptake assay using the fluorescent fatty acid analog BODIPY FL C16 (5 µM, 30 min at 37 °C) followed by flow cytometry analysis, showed no substantial increase in lipid uptake in dormant cells compared with proliferative or recurrent cells (Supplementary Fig. S4E‑H). Pharmacological inhibition of autophagy with Spautin-1 significantly suppressed LD accumulation (Fig. 5L-M and Supplementary Fig. S4I-J) [41], as quantified by flow cytometry. Consistently, genetic ablation of FIP200 phenocopied this suppression (Fig. 5N-Q and Supplementary Fig. S4K-L). These data collectively demonstrate that upregulated autophagy, not exogenous uptake, provides the principal lipids for droplet biogenesis in dormant tumor cells.

### The ELF1-ACSL5 axis mediates the dormant state of CRC cells

**Fig. 6:**
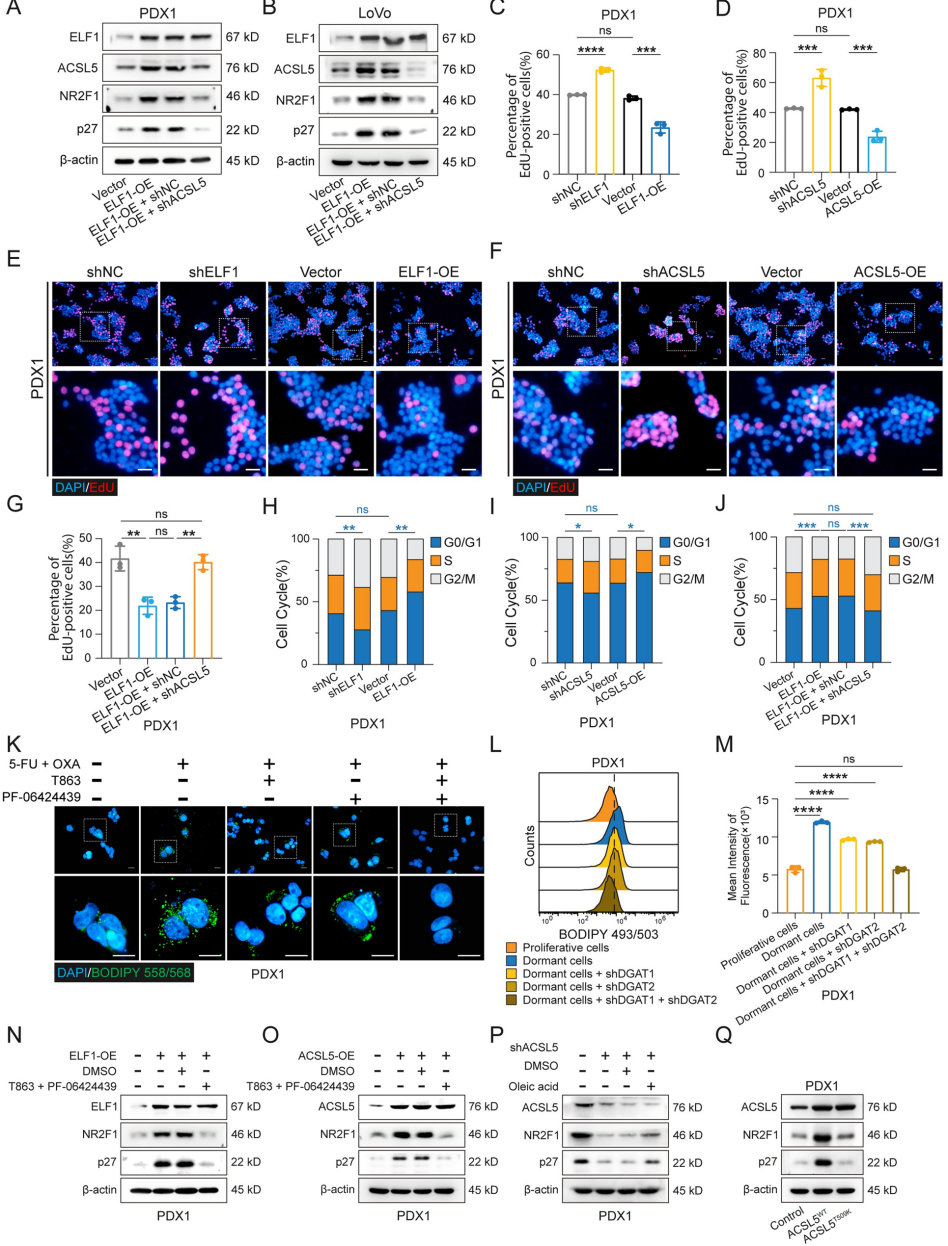
The ELF1-ACSL5 axis mediates the dormant state of CRC cells. **A**-**B** Western blot analysis of the dormancy markers in PDX1 or LoVo cells following ELF1 overexpression and/or ACSL5 knockdown. **C**-**G** EdU assays quantified the percentage of EdU-positive PDX1 cells following the indicated genetic perturbations. (n = 3). (E-F) Representative microphotographs. Scale bar: 20 μm. **H**-**J** Cell cycle analysis of PDX1 cells by flow cytometry under the indicated genetic perturbations (n = 3). **K** Representative microphotographs of the fluorescence analysis of lipid droplet content in dormant PDX1 tumor cells following treatment with the DGAT1 inhibitor T863, the DGAT2 inhibitor PF-06424439, or their combination. Scale bar: 20 μm. **L**-**M** Flow cytometric analysis of lipid droplet content in dormant PDX1 cells following individual or combined knockdown of DGAT1 and DGAT2. (M) The statistical analysis of the flow cytometry (n = 3). **N**-**O** Western blot analysis of NR2F1 and p27 expression in PDX1 cells under the indicated genetic and pharmacological perturbations. **P** Western blot analysis of NR2F1 and p27 expression in PDX1 cells upon knockdown of ACSL5 and addition of Oleic acid. **Q** Western blot analysis of dormancy markers in PDX1 cells overexpressing either ACSL5^WT^ or a catalytically inactive ACSL5 mutant (ACSL5^T509K^). Data are presented as mean ± SD, and statistical analysis was performed using one-way ANOVA. ns, no significance. *p < 0.05, **p < 0.01, ***p < 0.001, ****p < 0.0001

Dormant CRC cells exhibit significantly higher LDs content compared to proliferative cells, as previously demonstrated (Fig. 2C-D). To assess whether LD abundance regulates dormancy, we knocked down ACSL5 and observed decreased LD levels (Fig. 5J) accompanied by reduced expression of dormancy markers (Fig. 6A-B), linking LD accumulation to dormancy maintenance.

We next evaluated the functional role of the ELF1–ACSL5 axis in dormancy maintenance using EdU incorporation and cell cycle analysis. Knockdown of ELF1 or ACSL5 increased EdU^+^ cell frequency, while their overexpression decreased it (Fig. 6C-F and Supplementary Fig. S5A-D). Combined ELF1 overexpression and ACSL5 knockdown further enhanced proliferation (Fig. 6G). Flow cytometry confirmed that ELF1 or ACSL5 knockdown reduced G0/G1 arrest, whereas overexpression reinforced it—effects reversed by ACSL5 co-knockdown (Fig. 6H-J). These results establish ELF1 and ACSL5 as positive regulators of CRC dormancy, with ELF1 acting upstream.

To validate the coupling between LD biogenesis and dormancy, we inhibited DGAT1 and DGAT2—key ER-resident enzymes for LD synthesis—using pharmacological inhibitors (T863 and PF-06424439) [42, 43] or genetic ablation. Both approaches significantly suppressed LD accumulation (Fig. 6K-M and Supplementary Fig. S5E-J). Inhibitor treatment reduced dormancy markers even under ELF1 or ACSL5 overexpression (Fig. 6N-O and Supplementary Fig. S5K-L), confirming LD-dependent dormancy. We then supplemented ACSL5-knockdown cells with oleic acid to elevate LDs exogenously [44]. This restored NR2F1 and p27 expression (Fig. 6P and Supplementary Fig. S5M), effectively rescuing dormancy. Conversely, expression of a catalytically inactive ACSL5 mutant failed to promote dormancy (Fig. 6Q and Supplementary Fig. S5N). Collectively, the ELF1-ACSL5 axis sustains CRC dormancy by orchestrating LD accumulation, positioning LD metabolism as a therapeutic vulnerability.

### ACSL5 increased the lipid droplets content and thus decreased the intracellular ROS level, finally positively mediated the dormancy of the CRC cells

LD serve dual roles as energy reservoirs and metabolic regulators, storing fatty acid, promoting cell survival, and conferring resistance to oxidative stress through dynamic biogenesis [8, 14, 45]. Tumor cell dormancy depends on redox homeostasis, characterized by attenuated oxidative stress and suppressed reactive oxygen species (ROS) levels [13]. Astrocytes, for instance, compartmentalize oxidized lipids within LDs to mitigate oxidative damage, a mechanism conserved across stress-resistant cell types [14].

**Fig. 7:**
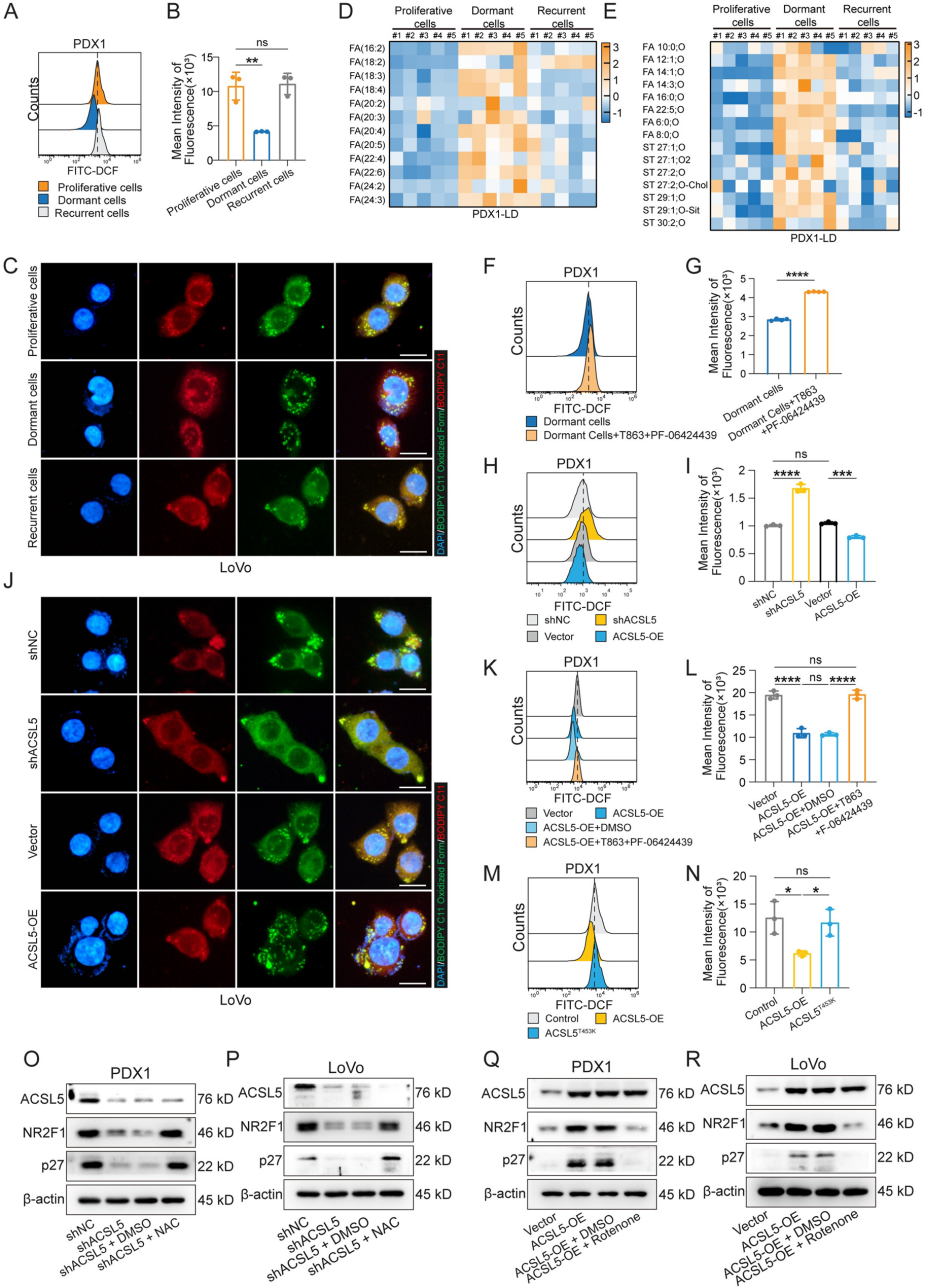
Lipid droplet accumulation driven by ACSL5 promotes CRCs dormancy through oxidized lipid sequestration. **A**-**B** ROS levels in proliferative, dormant and recurrent PDX1 cells (n = 3). C Subcellular localization of total lipids (BODIPY C11, red) and peroxidized lipids (BODIPY C11 Oxidized Form, green) in LoVo tumor cells. Upon oxidation, this probe exhibits a spectral shift in fluorescence emission from 590 nm (red) to 510 nm (green). Scale bar: 20 μm. **D**-**E** Heatmaps depicting the relative abundance of (**D**) polyunsaturated lipids and (**E**) oxidized lipids measured by lipidomics in isolated lipid droplets from proliferative, dormant and recurrent PDX1 cells. Lipid abundance was normalized to cell number (nmol per 10⁶ cells) and Z‑score standardized for visualization. Rows represent individual lipid species; columns represent biological replicates (#1–#5) for each state. Color intensity indicates relative lipid abundance (blue, low abundance; orange, high abundance). See Table S8 for details. **F**-**G** ROS levels in dormant PDX1 cells treated with T863 and/or PF-06424439 (n = 4). **H**-**I** ROS levels in PDX1 cells upon knockdown or overexpression of ACSL5 (n = 3). **J** Immunofluorescence analysis of peroxidized lipid distribution (BODIPY C11 Oxidized Form, green) in LoVo cells upon knockdown or overexpression of ACSL5. Scale bar: 20 μm. **K**-**L** ROS levels in PDX1 cells overexpressing ACSL5 and/or treated with T863 and PF-06424439 (n = 3). **M**-**N** Intracellular ROS levels in PDX1 cells expressing a catalytically inactive ACSL5 mutant (ACSL5^T509K^) (n = 3). **O**-**P** Western blot analysis of NR2F1 and p27 expression in PDX1 and LoVo cells following ACSL5 knockdown and/or NAC treatment. **Q**-**R** Western blot analysis of NR2F1 and p27 expression in PDX1 and LoVo cells following ACSL5 overexpression and/or rotenone treatment. Data are presented as mean ± SD. One-way ANOVA for (B, G, I, L and N). ns, no significance. *p < 0.05, **p < 0.01, ***p < 0.001, ****p < 0.0001

To assess ACSL5’s role in dormancy via oxidative stress modulation, we quantified ROS levels in dormant, recurrent, and proliferative CRC cells. Dormant cells exhibited significantly lower ROS than proliferative or recurrent counterparts (Fig. 7A-B and Supplementary Fig. S6 A-B). The endoplasmic reticulum is the primary site of lipid droplet biogenesis, and cells actively sequester peroxidized fatty acids into LDs via the ER to prevent their accumulation in other cellular membranes and to mitigate oxidative stress-induced damage [46]. This will reduce intracellular ROS generation and confer cyto-protection against ROS-mediated damage [45]. Using the lipid peroxidation sensor BODIPY C11 [47], we detected pronounced peroxidized lipid accumulation within LDs of dormant cells (Fig. 7C). Lipidomic profiling via LC–MS/MS showed a marked enrichment of unsaturated lipids in the LD fraction of dormant cells (Fig. 7D and E). Furthermore, we extracted lipid droplets, used a whole‑cell lipid fraction as a control, and performed the analysis according to rigorous lipidomics protocols. The purity of the extracted lipid droplets was verified by Western blotting, confirming the feasibility of the method (Supplementary Fig. S6C). As shown in Fig. 7D‑E, the levels of both oxidized and unsaturated lipids were higher in dormant tumor cells than in proliferative or recurrent cells. In the lipidomics analysis of whole‑cell lysates, the levels of both oxidized and unsaturated lipids showed no substantial differences among the three groups, which further supports the role of lipid droplets in sequestering these lipids (Supplementary Fig. S6D‑E). In addition to the fold‑change heatmap overview, we performed statistical analysis on individual lipid species presented in Supplementary Fig. S6F and Fig. S7A. Most of the displayed lipid species were more abundant in dormant cells, and even for those without statistical difference, a trend toward higher abundance observed in dormant cells. Pharmacological inhibition of LD biogenesis elevated intracellular ROS (Fig. 7F-G and Supplementary Fig. S8A-B). ACSL5 knockdown elevated ROS, while its overexpression suppressed ROS (Fig. 7H-I and Supplementary Fig. S8C-D). Consistent with these findings, fluorescence staining revealed that ACSL5 knockdown reduced LD content and dispersed oxidized lipids in the cytosol, whereas ACSL5 overexpression enhanced peroxidized lipid sequestration into LDs (Fig. 7J). ROS reduction induced by ACSL5 overexpression was abolished upon LD inhibition (Fig. 7K-L and Supplementary Fig. S8E-F). Catalytic inactivation of ACSL5 abrogated both ROS suppression and cytoprotection (Fig. 7M-N and Supplementary Fig. S8G-H).

To test ROS-dependent in dormancy, we modulated redox balance using N-acetylcysteine (NAC) [48–50] or the ROS inducer rotenone [51]. NAC rescued dormancy markers in ACSL5-knockdown cells (Fig. 7O-P), whereas rotenone disrupted ACSL5-mediated dormancy (Fig. 7Q-R). These results establish ACSL5 as a critical redox guardian that sustains dormancy by sequestering oxidized lipids into LDs. In summary, ACSL5 promotes dormancy in CRC by orchestrating LD-dependent redox balance, positioning LD-mediated ROS mitigation as a targetable mechanism to prevent recurrence.

### ACSL5 is positively associated with poor prognosis of the patients with CRC

**Fig. 8:**
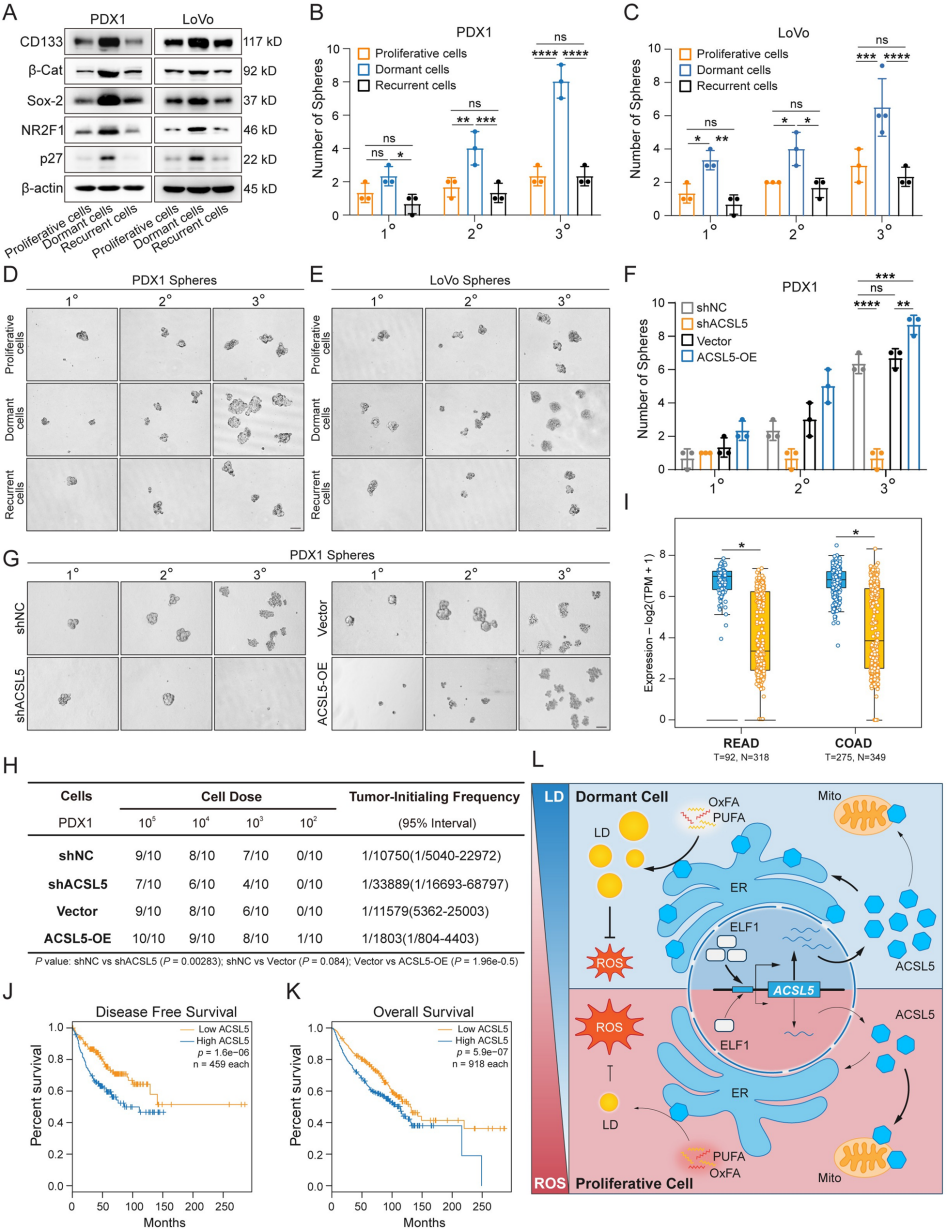
ACSL5 is positively associated with poor prognosis of the patients with CRC. **A** Western blot analysis of the expression of stemness-related genes in proliferative, dormant and recurrent PDX1 and LoVo cells. **B**-**E** Serial sphere formation assays for proliferative, dormant and recurrent PDX1 and LoVo cells (n = 3). Scale bar: 300 μm. **F**-**G** Serial sphere formation assays for PDX1 cells upon knockdown or overexpression of ACSL5 (n = 3). Scale bar: 300 μm. **H** Tumor-initiating frequency of PDX1 cells upon knockdown or overexpression of ACSL5 by extreme limiting dilution analysis (ELDA). **I** Analysis of ACSL5 expression in colorectal cancer tissues and normal tissues by TCGA. Comparisons were made using Student’s t-test. **J**-**K** The disease-free survival (DFS) and overall survival (OS) of the patients with CRC. The survival analysis is based on data from the TCGA. **L** Schematic diagram. ACSL5 upregulation in dormant tumor cells drives LD accumulation to reduce ROS and sustain dormancy. ELF1 binds the ACSL5 promoter, establishing an ELF1-ACSL5-LD axis critical for redox balance. Disrupting this axis depletes LDs, elevates ROS, and forces dormancy exit, linking organelle metabolism to tumor dormancy regulation. LD, lipid droplet; FFA, free fatty acid; Mito, mitochondria; ER, endoplasmic reticulum; ROS, Reactive oxygen species. Data are presented as mean ± SD. Statistical analysis was performed using one-way ANOVA in (**B**-**C, F**). ns, no significance. *p < 0.05, **p < 0.01, ***p < 0.001, ****p < 0.0001

Dormant tumor cells are enriched for cancer stem cell populations [52–54], prompting investigation of stemness features in dormant CRC cells within our model. Western blot analysis and sphere formation assays were performed across proliferative, dormant, and recurrent CRC cells. Dormant cells exhibited elevated expression of stemness markers (e.g., SOX2, CD133) compared to proliferative or recurrent populations (Fig. 8A) and demonstrated enhanced sphere-forming capacity (Fig. 8B-E). Relative to proliferative and recurrent tumor cells, dormant cells showed increased N‑cadherin expression and decreased E‑cadherin expression, whereas ACSL5 knockdown reversed this expression pattern (i.e., reduced N‑cadherin and elevated E‑cadherin) (Supplementary Fig. S9A‑B).

To interrogate ACSL5’s role in stemness regulation, we conducted sphere formation and limiting dilution assays (LDAs). ACSL5 knockdown reduced sphere formation, while its overexpression amplified it (Fig. 8F-G). LDAs revealed that ACSL5 knockdown impaired tumor-initiating capacity, whereas overexpression enhanced it (Fig. 8H). As shown in Supplementary Fig. S9C-I, ACSL5-knockdowning cells developed larger tumors. In contrast, ACSL5-overexpressing cells, which are prone to dormancy, required a longer time to exit the dormant state and consequently formed smaller tumors. Regarding tumor incidence, given their enhanced stemness and tumor-initiating capacity, dormant cells produced more tumors. TCGA analysis confirmed elevated ACSL5 expression in CRC tumors compared to normal colorectal tissues (Fig. 8I). High ACSL5 expression correlated with reduced disease-free survival (DFS) and overall survival (OS) in CRC patients (Fig. 8J-K), consistent with our preclinical data showing ACSL5-driven tumorigenicity. Collectively, ACSL5 emerges as a biomarker of aggressive CRC, mechanistically linking lipid metabolism to stemness and poor clinical outcomes.

## Discussion

Although metabolic reprogramming is a critical adaptation in dormant tumor cells, the contribution of organelle-centric metabolism—particularly lipid droplet (LD) biogenesis—to dormancy maintenance remains poorly defined. Using patient-derived xenografts and colorectal cancer (CRC) cell lines, we identify the ELF1-ACSL5 axis as a key regulator of LD accumulation in dormant CRC cells. We show that ACSL5-dependent LD formation reduces intracellular reactive oxygen species (ROS), thereby promoting dormancy. Mechanistically, the transcription factor ELF1 directly binds the ACSL5 promoter, connecting transcriptional regulation to organelle metabolism. These results reveal a previously unrecognized LD-mediated redox mechanism for dormancy stabilization and nominate the ELF1-ACSL5-LD axis as a therapeutic target to prevent recurrence.

Dormant tumor cells (DTCs) persist long after therapy and drive recurrence through quiescence, niche interactions, and immune evasion. Their dormant phenotype is sustained through cell cycle arrest (e.g., p38/p21), dynamic interactions with specialized microenvironments (e.g., bone marrow stromal niches), and immune evasion mechanisms [20]. Our dormant cell model, induced by chemotherapy (5-FU + oxaliplatin), recapitulates several key features commonly associated with cancer cell dormancy in colorectal cancer, as reported in previous studies: (1) upregulation of dormancy markers, including NR2F1 and p27 [20, 24]; (2) the ability to survive chemotherapy, which mimics the clinical scenario of minimal residual disease (MRD) following adjuvant therapy [55]; and (3) the capacity to re-enter the cell cycle upon withdrawal of chemotherapeutic stress [20, 56]. Emerging research underscores epigenetic reprogramming—including DNA hypermethylation and miRNA-mediated silencing—and metabolic adaptations (e.g., suppressed oxidative phosphorylation) as pivotal regulators of DTC survival [57, 58]. Tumor stem-like cells (CSCs) further reinforce dormancy by activating autophagy and self-renewal pathways (e.g., Wnt/Notch), enabling long-term persistence [58, 59]. Emerging therapies targeting dormancy-awakening checkpoints, including neutrophil extracellular traps (NETs) and epigenetic modulators [60, 61], offer promising strategies to prevent relapse. Deciphering the spatiotemporal dynamics of these interactions is critical for disrupting metastatic relapse.

DTCs display metabolic plasticity under stress conditions, often accumulating LDs—a hallmark of chemoresistance [20]. Beyond energy storage, LDs regulate cellular homeostasis, buffer lipotoxic stress, and support membrane biosynthesis [8, 29, 62]. Hypoxia and nutrient deprivation in the tumor microenvironment (TME) further drive LD biogenesis via HIF-1α and sterol regulatory element-binding protein (SREBP)-dependent lipogenic pathways [63–66]. Emerging evidence positions LDs as signaling hubs that modulate oncogenic pathways, promoting chemoresistance and immune evasion [67, 68]. Residual LD-enriched cancer cells exhibit enhanced post-therapy survival, with chemotherapy or radiation stress upregulating lipases (e.g., ATGL, HSL) to liberate fatty acids (FAs) for mitochondrial ATP synthesis and redox homeostasis [69–71]. LD-driven metabolism also activates pro-survival autophagy and suppresses ferroptosis, a lipid peroxidation-dependent cell death mechanism [10, 72–74]. Notably, LD-rich CSCs utilize these pathways to maintain stemness and facilitate recurrence, with elevated LD levels correlating with poor prognosis in several cancers [75, 76].

Dormant tumor cells maintain quiescence by sustaining low reactive oxygen species (ROS) levels via antioxidant defenses such as NRF2 signaling [9]. We demonstrate that ACSL5-mediated LD accumulation reduces intracellular ROS, thereby stabilizing colorectal cancer (CRC) dormancy. The ELF1-ACSL5 axis mechanistically governs LD biogenesis in dormant CRC cells, linking lipid metabolism to dormancy maintenance. ACSL5, a context-dependent regulator of lipid metabolism [29, 76], catalyzes acyl-CoA ester formation to influence lipid storage and energy homeostasis, suggesting its role in dormancy via metabolic adaptation [77]. ACSL5 overexpression in CRC correlates with chemoresistance and dormant cell survival, enabling FA storage during nutrient deprivation or hypoxia [78]. ELF1, an ETS-family transcription factor, orchestrates DNA repair, epithelial-mesenchymal transition (EMT), and cell cycle regulation [79]. In osteosarcoma, ELF1 transcriptionally activates FOXD3-AS1, driving metastasis via a miR-296-5p/ZCCHC3 ceRNA axis [79]. In CRC dormancy, ELF1 transcriptionally controls quiescence and stress resistance while directly activating oncogenes (e.g., CCND1, MMP9) and repressing tumor suppressors (e.g., CDKN1A) [40, 62–64, 80].

Our study has limitations inherent to the complexity of LDs—organelles with multifaceted roles in cellular metabolism, stress adaptation, and signaling. While we elucidate LD-driven redox regulation and dormancy maintenance in colorectal cancer (CRC), their broader functional repertoire across biological contexts remains underexplored. Our current in vivo experiments reflect tumor cell dormancy indirectly through differences in tumor size, which correspond to the timing of proliferation onset. Future studies using chemotherapy-induced dormant tumor cells will be necessary to directly examine whether ACSL5-mediated lipid droplet formation is required for the maintenance of the dormant state in vivo. Advanced models (e.g., patient-derived organoids, in vivo tracing of LD dynamics) and cutting-edge techniques (single-cell lipidomics, CRISPR-Cas9 screens) will help resolve spatiotemporal LD heterogeneity and their interplay with oncogenic pathways. Additionally, translating these findings into clinical applications will require validating LD-associated targets (e.g., ACSL5, DGAT1) in diverse tumor types and metastatic niches. By integrating mechanistic insights with therapeutic innovation, this work opens avenues to disrupt dormancy and prevent recurrence through LD-centric strategies.

## Conclusions

Here we show that LD accumulation, governed by the ELF1-ACSL5 axis, sustains tumor dormancy through a redox-regulatory mechanism. By sequestering oxidized lipid and mitigating oxidative stress, LD accumulation enforces a dormant, chemoresistant state in colorectal cancer cells. These findings position organelle-centric lipid metabolism as a critical vulnerability of dormant colorectal cancer cells. Targeting the ELF1-ACSL5-LD pathway thus represents a promising therapeutic strategy to perturb dormancy and prevent metastatic relapse.

## Supplementary Information

Supplementary Material 1.

## Data Availability

Availability of data and materialsThe data that support the findings of this study are available from the corresponding author upon reasonable request.

## Abbreviations

5-Fu: 5-Fluorouracil
ACS: Acyl-CoA Synthetase
ACSL5: Acyl-CoA Synthetase Long-Chain Family Member 5
ACSL6: Acyl-CoA Synthetase Long-Chain Family Member 6
ACSBG1: Acyl-CoA Synthetase Bubblegum Family Member 1
ACSBG2: Acyl-CoA Synthetase Bubblegum Family Member 2
ATCC: American Type Culture Collection
BCA: Bicinchoninic Acid Assay
BSA: Bovine Serum Albumin
C/EBP: α-CCAAT/Enhancer-Binding Protein-αpha
CCK: 8-Cell Counting Kit-8
CD36: Cluster of Differentiation 36
CD133: Cluster of Differentiation 133
CRC: Colorectal Cancer
DAPI: 4’,6-diamidino-2-phenylindole
DGAT1: Diacylglycerol Acyltransferase 1
DGAT2: Diacylglycerol Acyltransferase 2
DMEM: Dulbecco’s Modified Eagle Medium
DEGs: Differentially Expressed Gene
EDTA: Ethylenediaminetetraacetic acid
EdU: 5-Ethynyl − 2’-deoxyuridine
ELDA: Extreme Limiting Dilution Analysis
ELF1: E74 Like ETS Transcription Factor 1
ER: Endoplasmic Reticulum
FIP200: RB1-inducible coiled-coil protein 1
FBS: Fetal Bovine Serum
GEPIA2: Gene Expression Profiling Interactive Analysis 2
LC: MS/MS-Liquid Chromatography-Tandem Mass Spectrometry
LD: Lipid Droplets
Mito: Mitochondria
NAC: N-Acetyl-L-Cysteine
NR2F1: nuclear receptor subfamily 2, group F, member 1
ORO: Oil Red O
OXA: Oxaliplatin
p27: Cyclin Dependent Kinase Inhibitor 1B
p62: Sequestosome 1
PBS: Phosphate-Buffered Saline
PCC: Pearson Correlation Coefficient
PDI: Protein Disulfide Isomerase
PDX1: Patient derived xenograft-1
PFA: Paraformaldehyde
PI: Propidium Iodide
PVDF: Polyvinylidene Difluoride
qPCR: quantitative Polymerase Chain Reaction
ROS: Reactive Oxygen Species
SDS: PAGE-Sodium Dodecyl Sulfate-Polyacrylamide Gel Electrophoresis
SOX2: SRY-box 2
SPF: Specific Pathogen Free
TFs: Transcription Factors
TOM20: Translocase of the Outer Mitochondrial membrane 20
β-Catenin: Catenin beta-1
