# Supplementary material for: Intracellular lipid droplets positively regulate the dormancy of colorectal cancer cells depending on ELF1-ACSL5 signaling
Source: J Exp Clin Cancer Res. 2026 Jun 11;45:181. doi: 10.1186/s13046-026-03754-y (PMC13483860; doi:10.1186/s13046-026-03754-y)
Supplement: Supplementary file 1 [file 13046_2026_3754_MOESM1_ESM.docx]

Supporting Information

Title

**Intracellular lipid droplets positively regulate the dormancy of colorectal cancer cells depending on ELF1-ACSL5 signaling**

*Wancheng Liao^1,2^, Rui Yin^1,2^, Fan Zuo^1,2^, Yangkun Li^1,2,3^, Jiao Deng^1,2^, Yanqi Li^1,2^, Xiaolan Li^1^, Deding Tao^1^, Xuelai Luo^1,2^ Weike Ji^4,5^* and Jichao Qin^1,2,3^**

**Supplementary Fig. 1. Related to Fig. 1&2.**

**A** CCK-8 assay of cell viability (OD 450 nm) in LoVo cells treated with the indicated doses of 5-Fu and OXA for 96 hours (n=3). **B** Quantification of NR2F1⁺/LD⁺ double‑positive cancer cells in post‑chemotherapy tumors (n = 4) (corresponding to Fig. 2I). Data are presented as mean ± SD. All error bars in all figures represent the mean ± SD unless otherwise stated. Statistical analysis was performed using two-way ANOVA in (A), and one-way ANOVA in (B). ****p < 0.0001.

**Supplementary Fig. 2. Related to Fig. 3.**

**A** Volcano plots displaying differential gene expression in recurrent vs dormant PDX1 cells. **B** Heatmap depicting fold changes in expression of altered genes within the fatty acid biosynthesis pathway (KEGG: hsa00061) in proliferative, dormant and recurrent CRC cells. Data were derived from RNA‑seq analysis (n = 3). **C-F** Quantitative real-time PCR (qPCR) analysis of *ACSL5*, *ACSL6*, *ACSBG1* and *ACSBG2* mRNA in proliferative, dormant and recurrent PDX1 cells. n = 3 biologically independent samples in **(C)**; n = 5 in **(D)**; n = 4 in **(E-F)**. **G** Western blot analysis of ACSL6, ACSBG1 and ACSBG2 in proliferative, dormant and recurrent PDX1 and LoVo cells. **H** Representative microphotographs of the Immunofluorescence staining for ACSL5 (red) and mitochondria (TOM20, green) in LoVo cells. Scale bar: 20 μm. **I** Quantification of the co-localization between ACSL5 and mitochondria from the fluorescence images in Supplementary Fig. S2H. Pearson's correlation coefficient was used to assess the degree of co-localization in proliferative, dormant, and recurrent LoVo cells (n = 4). **J-K** Western blot analysis of isolated endoplasmic reticulum (ER) and mitochondrial (Mito) fractions using organelle-specific markers. GAPDH (cytoplasm), Lamin B1 (nucleus), VDAC1 (Mito), and PDI (ER). **L-M** Uncropped original blot images corresponding to the membranes shown in Fig. 3**G-H**. Data are presented as mean ± SD. Statistical analysis was performed using one-way ANOVA in **(C-F and I)**. ns, not significant. ***p < 0.001, ****p < 0.0001.

**Supplementary Fig. 3. Related to Fig. 3.**

**A-B** Western blot analysis of ACSL5 expression in PDX1 and LoVo cells following knockdown with shACSL5 (shNC as negative control) and overexpression with ACSL5-OE vector. **C-D** The lipid droplets were measured in dormant LoVo cells upon knockdown or overexpression of ACSL5 by flow cytometry (n = 3). **E** Representative microphotographs of immunofluorescence staining to observe the lipid droplets in dormant LoVo cells upon knockdown or overexpression of ACSL5. Scale bar: 20 μm. **F-G** The statistical analysis of the abundance or area of lipid droplets in Supplementary Fig. 3E (n = 3). **H-K** Flow cytometric analysis of lipid droplet content in dormant PDX1 and LoVo cells following inhibition of ACSL5 with Triacsin C. **(I and K)** The statistical analysis of the flow cytometry (n=3). **L** Western blot analysis confirming expression levels of wild-type ACSL5 (ACSL5^WT^) and the catalytic dead point mutant (ACSL5^T509K^) in LoVo cells. **M** ACS enzymatic activity assay measuring acyl-CoA synthetase (ACS) activity in cells expressing vector control, ACSL5^WT^, and ACSL5^T509K^ in LoVo cells (n=3). Activity is shown in mU/ml. **N-O** Flow cytometric analysis of lipid droplet content in tumor cells expressing vector control, ACSL5^WT^, and ACSL5^T509K^ in LoVo cells. **(O)** The statistical analysis of the flow cytometry (n=3). Data are presented as mean ± SD. Statistical analysis was performed using one-way ANOVA in **(D, F-G, I, K, M and O)**. ns, not significant. *p < 0.05, **p < 0.01, ***p < 0.001, ****p < 0.0001.

**Supplementary Fig. 4. Related to Fig. 5.**

**A** The statistical analysis of the flow cytometry in Fig. 5**J**. **B-C** The lipid droplets were examined by flow cytometry in LoVo cells overexpressed ELF1 and/or knocked down ACSL5. **(C)** The statistical analysis of the flow cytometry (n = 3). **D** Heatmap depicting expression changes of selected lipid transporters (identified by RNA‑seq) in proliferative, dormant and recurrent CRC cells. Data were derived from RNA‑seq analysis (n = 3). The color scale represents log₂‑transformed fold change. **E‑H** Lipid uptake assays. Proliferative, dormant and recurrent PDX1 and LoVo cells were incubated with the fluorescent fatty acid analog BODIPY FL C16 (5 μM, 30 min at 37 °C). Cellular lipid uptake was quantified by flow cytometry. **I-J** Flow cytometric analysis of lipid droplet content in dormant LoVo cells following autophagy inhibition with Spautin-1. **(J)** The statistical analysis of the flow cytometry (n = 3). **K-L** Western blot validation of knockdown efficiency for the key autophagy regulator FIP200 in PDX1 or LoVo cells. Data are presented as mean ± SD, and statistical analysis was performed using one-way ANOVA. ns, not significant. *p < 0.05, **p< 0.01, ***p < 0.001, ****p < 0.0001.

**Supplementary Fig. 5. Related to Fig. 6.**

**A-D** EdU assays quantified the percentage of EdU-positive LoVo cells following the indicated genetic perturbations. (n = 3). Scale bar: 20 μm. **E-H** Western blot validation of DGAT1 or DGAT2 knockout efficiency in PDX1 and LoVo cells. **I-****J** Flow cytometric analysis of lipid droplet content in dormant LoVo cells following individual or combined knockdown of DGAT1 and DGAT2. **(J)** The statistical analysis of the flow cytometry (n = 3). **K-L** Western blot analysis of NR2F1 and p27 expression in LoVo cells under the indicated genetic and pharmacological perturbations. **M** Western blot analysis of NR2F1 and p27 expression in LoVo cells upon knockdown of ACSL5 and addition of Oleic acid. **N** Western blot analysis of dormancy markers in LoVo cells overexpressing either ACSL5^WT^ or a catalytically inactive ACSL5 mutant (ACSL5^T509K^). Data are presented as mean ± SD, and statistical analysis was performed using one-way ANOVA. ns, not significant. ***p < 0.001, ****p < 0.0001.

**Supplementary Fig. 6. Related to Fig. 7.**

**A-B** Relative levels ROS were measured in proliferative, dormant and recurrent LoVo cells. **(B)** The statistical analysis of the flow cytometry (n = 3). **C** Purity validation of lipid droplet. Western blotting analysis of organelle‑specific markers in isolated lipid droplet (LD), whole‑cell lysate (WCL), and LD‑depleted fractions. PDI (endoplasmic reticulum marker), VDAC1 (mitochondrial marker), lamin B1 (nuclear marker), and GAPDH (cytoplasmic marker) were probed. The LD fraction showed minimal contamination from other organelles. **D‑E** Heatmaps depicting the relative abundance of polyunsaturated lipids (D) and oxidized lipids (E) measured by lipidomics in whole‑cell lysates from proliferative, dormant, and recurrent PDX1 cells. Lipid abundance was normalized to cell number (nmol per 10⁶ cells) and Z‑score standardized for visualization. Rows represent individual lipid species; columns represent biological replicates (#1–#5) for each cell state. Color intensity indicates relative lipid abundance (blue, low; orange, high). **F** Statistical analysis of the absolute abundance (nmol per 10⁶ cells) of each polyunsaturated lipid species shown in Fig. 7D (n = 3). Data are presented as mean ± SD. Statistical analysis was performed using one-way ANOVA in **(F)**. ns, not significant. *p < 0.05, **p < 0.01, ***p < 0.001, ****p < 0.0001.

**Supplementary Fig. 7. Related to Fig. 7.**

**A** Statistical analysis of the absolute abundance (nmol per 10⁶ cells) of each oxidized lipid species shown in Fig.7E (n = 3). Data are presented as mean ± SD. Statistical analysis was performed using one-way ANOVA in **(A)**. ns, no significant difference. *p < 0.05, **p < 0.01, ***p < 0.001, ****p < 0.0001.

**Supplementary Fig. 8. Related to Fig. 7.**

**A-B** Relative levels ROS were measured in dormant LoVo cells, with or without treatment of T863 and PF-06424439. **(B)** The statistical analysis of the flow cytometry (n = 4). **C-D** Relative levels ROS were measured in LoVo cells upon knockdown or overexpression of ACSL5. **(D)** The statistical analysis of the flow cytometry (n = 3). **E-F** Relative levels ROS were measured in LoVo cells upon overexpression of ACSL5 and/or treatment of T863 and PF-06424439. **(F)** The statistical analysis of the flow cytometry (n = 3). **G-H** Measurement of intracellular reactive oxygen species (ROS) levels in LoVo tumor cells expressing a catalytically inactive ACSL5 mutant (ACSL5^T509K^). **(H)** The statistical analysis of the flow cytometry (n = 3). Data are presented as mean ± SD. Statistical analysis was performed using one-way ANOVA in **(B, D, F and H)**. ns, not significant. *p < 0.05, **p < 0.01, ***p < 0.001, ****p < 0.0001.

**Supplementary Fig. 9. Related to Fig. 8.**

**A** Western blot analysis of E‑cadherin and N‑cadherin expression in proliferative, dormant, and recurrent PDX1 and LoVo cells. **B** Western blotting analysis of E‑cadherin and N‑cadherin expression in PDX1 and LoVo cells upon knockdown of ACSL5 (shACSL5). **C** Representative images of subcutaneous tumors derived from PDX1 cells injected at the indicated cell doses (corresponding to Fig. 8H). Scale bar: 1 cm. **D‑I** Quantitative analysis of subcutaneous tumor size and volume across experimental groups (shNC, shACSL5, vector, ACSL5‑OE) at various cell doses. (**D, G**) 1 × 10⁵ cells; (**E, H**) 1 × 10⁴ cells; (**F, I**) 1 × 10³ cells. For each cell dose, endpoint tumor weights are shown in (**D‑F**) and tumor volumes (mean ± SD) are shown in (**G‑I**). Statistical analysis was performed using one‑way ANOVA. *p < 0.05, **p < 0.01, ***p < 0.001; ns, not significant.

**Table**

Table S1. The information of the patient involved in the construction of PDX1 cells.

| **Patient** | **Gender/Age** | **TNM** | **Stage** |
| --- | --- | --- | --- |
| XhCRC-1 | F/47 | T4N2M0 | Ⅲ |

Table S2. Drugs, antibodies, and kits used in this study.

| REAGENT or RESOURCE | SOURCE | IDENTIFIER |
| --- | --- | --- |
| Antibodies | | |
| CD133 | Cell Signaling Technology | Cat#64326 |
| β-Catenin | Cell Signaling Technology | Cat#8480 RRID: AB_11127855 |
| Sox-2 | Cell Signaling Technology | Cat#3579 RRID: AB_2195767 |
| NR2F1 | Abcam | Cat#ab181137 RRID: AB_2890250 |
| p27 | MedChem Express | Cat#HY-P80256 |
| ACSL5 (A-2) | Santa Cruz Biotechnology | Cat#sc-365478 RRID: AB_10847089 |
| ACSL5 | Proteintech | Cat#15708-1-AP RRID: AB_2257881 |
| ACSBG1 | Proteintech | Cat#16077-1-AP RRID: AB_2878214 |
| ACSBG2 | Proteintech | Cat#19083-1-AP RRID: AB_3085573 |
| DGAT2 | Proteintech | Cat#17100-1-AP RRID: AB_2918049 |
| PDI | Proteintech | Cat#66422-1-Ig |
| ACSL6 | Abclonal | Cat#A7965 RRID: AB_2768228 |
| VDAC1 | Abclonal | Cat#A19707 RRID: AB_2862746 |
| β-actin | Abclonal | Cat#AC026 RRID: AB_2768234 |
| TOM20 | Abclonal | Cat#A19403 RRID: AB_2862646 |
| p62 | Abclonal | Cat#A19700 RRID: AB_2862742 |
| ELF1 | Affinity Biosciences | Cat#AF0483 RRID: AB_2834232 |
| FIP200 | Huaan Biotechnology | Cat#HA601106 |
| CD36 | Huaan Biotechnology | Cat#ET1701-24 |
| DGAT1 | Huaan Biotechnology | Cat#HA721555 |
| Chemicals, Peptides, and Recombinant Proteins | | |
| 5-Fluorouracil | MedChem Express | Cat#HY-90006 |
| Oxaliplatin | MedChem Express | Cat#HY-17371 |
| T863 | MedChem Express | Cat#HY-32219 |
| PF-06424439 | MedChem Express | Cat#HY-108341A |
| Oleic acid | MedChem Express | Cat#HY-N1446 |
| Rotenone | MedChem Express | Cat#HY-B1756 |
| N-Acetylcysteine | MedChem Express | Cat#HY-B0215 |
| Triacsin C | MedChem Express | Cat#HY-N6707 |
| Spautin-1 | MedChem Express | Cat#HY-12990 |
| Ampicillin | MedChem Express | Cat#HY-B0522 |
| Kanamycin | MedChem Express | Cat#HY-16566 |
| Propidium Iodide | MedChem Express | Cat#HY-D0815 |
| BODIPY 581/591 C11 | Thermo Fisher Scientific | Cat#D3861 |
| BODIPY 493/503 | MedChem Express | Cat#HY-W090090 |
| BODIPY 558/568 C12 | Cayman Chemical | Cat# 158757-84-7 |
| DAPI | Sigma-Aldrich | Cat#d9542 |
| Puromycin | Thermo Fisher | Cat#A1113803 |
| Penicillin-Streptomycin | Gibco | Cat#15140122 |
| DMEM | Gibco | Cat#11965082 |
| FBS | Gibco | Cat#10099158 |
| BSA | Beyotime | Cat#ST023 |
| B27 | Gibco | Cat#A1486701 |
| Human EGF | Sigma-Aldrich | Cat#SRP3027 |
| Human basic-FGF | Gibco | Cat#PHG0264 |
| Opti-MEM | Gibco | Cat#31985070 |
| HighGene Plus Transfection Reagent | ABclonal | Cat#RM09014P |
| Critical commercial assays | | |
| Oil Red O Staining Kit | Leagene Biotechnology | Cat#DL0008 |
| BCA Assay Kit | Thermo Scientific | Cat#A55864 |
| Cell Counting Kit-8 | MedChemExpress | Cat#HY-K0301 |
| EdU Cell Proliferation Image Kit | Abbkine | Cat#KTA2031 |
| Endoplasmic Reticulum Extraction Kit | Solarbio | Cat#EX1370 |
| Mitochondria Isolation Kit | Beyotime | Cat#C3601 |
| Dual-Luciferase Reporter Assay Kit | Beyotime | Cat#RG027 |
| Amplex Red Acetyl-CoA Synthetase Assay Kit | Beyotime | Cat#S0391S |
| Reactive Oxygen Species Assay Kit | Beyotime | Cat#S0033 |
| Lipid Droplet Blue Fluorescence Detection Kit (LipiD-Blue) | Beyotime | Cat#C2052 |
| TaKaRa MiniBEST Universal Genomic DNA Extraction Kit | Takara Bio Inc. | Cat#9765 |
| Mut Express Universal Fast Mutagenesis Kit | Vazyme | Cat#C216-01 |
| ClonExpress Ultra One Step Cloning Kit | Vazyme | Cat#C116 |
| Lipid Droplet Isolation Kit | Cell Biolabs | Cat#MET-5011 |
| Bacterial and virus strains | | |
| pGL3-Basic vector | Addgene | Cat#48743 |
| pRL-TK plasmid | Beyotime | Cat#D2760 |
| psPAX2 | Addgene | Cat#12260, RRID: Addgene_12260 |
| pMD2.G | Addgene | Cat#12259, RRID: Addgene_12259 |
| Experimental models: Cell lines | | |
| LoVo | ATCC | Cat#CCL-229, RRID: CVCL_0399 |
| HEK293T | ATCC | Cat#CRL-3216, RRID: CVCL_0063 |
| Experimental models: Organisms/strains | | |
| NOD/SCID mice | GemPharmatech | Cat#T001492 |
| DH5α Chemically Competent *E*. coli | TSINGKE | Cat#TSC-C14 |
| Software and algorithms | | |
| FACSDiva software |  | BD Biosciences |
| FlowJo |  | BD Biosciences |
| SnapGene |  | Dotmatics |
| GraphPad Prism |  | Dotmatics |

Table S3. The primers used to obtain the promoter fragment of ACSL5

| Forward Primer | CGTTACATAACTTACGG |
| --- | --- |
| Reverse Primer | AGCTCTGCTTATATAGACC |

Table S4. The primers used to construct the plasmids in Dual luciferase assay

| Forward Primer-1 | GGCATTCCGGTACTGTTGGT |
| --- | --- |
| Reverse Primer-1 | AGCTCGGTACCTATCGATAGAGA |
| Forward Primer-2 | GATAGGTACCGAGCTAGGGTTTGTGAATGCACCAATCG |
| Reverse Primer-2 | CAGTACCGGAATGCCCTGTATTGATTCAGCACCACATTC |

Table S5. The information of the patient involved in immunofluorescence assay.

| **Patient** | **Gender/Age** | **TNM** | **Stage** | **Chemotherapy** |
| --- | --- | --- | --- | --- |
| 1 | M/57 | T4N1M1 | Ⅳ | Yes |
| 2 | F/64 | T4N2M1 | IV | Yes |
| 3 | F/54 | T3N1M1 | IV | Yes |
| 4 | F/44 | T3N2M1 | IV | Yes |
| 5 | M/52 | T4N1M0 | III | No |
| 6 | F/66 | T3N0M0 | II | No |
| 7 | F/65 | T3N2M1 | IV | No |

Table S6. The sequences of the primers for the quantitative RT-PCR.

| Gene | Forward Primer | Reverse Primer |
| --- | --- | --- |
| ACSL5 | TGGCTATCTTACAAACAGGTGTC | TCCACTCTGGCCTATTCTGAG |
| ACSL6 | GCACGGCGATCTGTGATTG | GGCGGAACACCTGGTACAT |
| ACSBG1 | CCCCGGAGTGGTTCTTCTC | CATAAGCGATGTACTGGCAGG |
| ACSBG2 | CCTTCTGAGGCTATCCAAACAC | CGGTTGACTGACTCTCGAAAAA |
| GAPDH | GGAGCGAGATCCCTCCAAAAT | GGCTGTTGTCATACTTCTCATGG |

Table S7. The sequences of the primers for site-directed mutagenesis

| Forward Primer | CACAGGTGGCTGTAAATTTACATTACCTGGGGACTGGAC |
| --- | --- |
| Reverse Primer | ATTTACAGCCACCTGTGCATTCTGTTTGACCA |

Table S8. Sterol species identified in Fig. 7E.

| Abbreviation | Lipid Species |
| --- | --- |
| FA 10:0;O | FA 10:0;O_2-hydroxy capric acid |
| FA 12:1;O | FA 12:1;O |
| FA 14:1;O | FA 14:1;O |
| FA 14:3;O | FA 14:3;O |
| FA 16:0;O | FA 16:0;O_2-hydroxy palmitic acid |
| FA 22:5;O | FA 22:5;O |
| FA 6:0;O | FA 6:0;O_2-hydroxy caproic acid |
| FA 8:0;O | FA 8:0;O_2-hydroxy caprylic acid |
| ST 27:1;O | ST 27:1;O_Cholesterol |
| ST 27:1;O2 | ST 27:1;O2_7-Hydroxy-5-cholestan-3-one |
| ST 27:2;O | ST 27:2;O_3-ketocholesterol |
| ST 27:2;O-Chol | ST 27:2;O-Cholestenone |
| ST 29:1;O | ST 29:1;O_23-methylene-24-methylcholestanol |
| ST 29:1;O-Sit | ST 29:1;O_Sitosterol |
| ST 30:2;O | ST 30:2;O_Verongulasterol_1 |
